# The mechanisms by which hypothalamic neuroinflammation induced by neonatal cerebral ischemia–hypoxia leads to decreased thymic function via the HPA axis

**DOI:** 10.1186/s13578-026-01543-w

**Published:** 2026-02-23

**Authors:** Gai-Gai Liu, Li-Yan Shuang, Qian Zhang, Guang-Jun Su, Yun Huang, Jin-Hua Xue, Li-Xia Jiang, Cheng Huang, Tao Chen, Zhi-Hua Huang, Si Cao

**Affiliations:** 1 Jiangxi Province Key Laboratory of Pharmacology of Traditional Chinese Medicine, Key Laboratory of Prevention and Treatment of Cardiovascular and Cerebrovascular Diseases of Ministry of Education, Ganzhou Key Laboratory of Neuroinflammation Research, School of Basic Medical Sciences, Gannan Medical University, Ganzhou, 341000 China; 2 Key Laboratory of Prevention and Treatment of Cardiovascular and Cerebrovascular Diseases of Ministry of Education, Ganzhou Key Laboratory of Neuroinflammation Research, First Affiliated Hospital of Gannan Medical University, Ganzhou, 341000 China; 3 The First People’s Hospital of Fuzhou City, Fuzhou, 344000 China; 4 School of Public Health and Health, Xinyu University, Xinyu, 338000 China

**Keywords:** Neonatal hypoxic–ischemic encephalopathy, Thymus function, HPA axis, Neuroinflammation

## Abstract

Hypoxic–ischemic encephalopathy (HIE) represents the leading cause of acute neonatal mortality and chronic neurological disorders. The impact of HIE on neonatal immune system development and the underlying mechanisms remain unclear. In this study, we employed the Sprague–Dawley neonatal rat hypoxic–ischemic (HI) model to investigated how HIE affected thymus and hypothalamic–pituitary–adrenal (HPA) axis, and to explore the mechanisms underlying their interaction. Our findings indicate that HI induced atrophy, elevated protein levels associated with apoptosis and autophagy, and reduced thymic lymphocyte counts in neonatal rats. Concurrently, HI suppressed activation of the PI3K/Akt/mTOR signaling pathway in thymus. Furthermore, HI significantly increased levels of inflammatory cytokines, activated microglia, and stimulated the HMGB1/TLR4/NF-κB signaling pathway in hypothalamus. HI also led to hyperactivation of the HPA axis. Conversely, treatment with the glucocorticoid receptor antagonist (RU486) or the HMGB1 inhibitor markedly attenuated these effects. These results suggest that HI may trigger neuroinflammation via activation of HMGB1/TLR4/NF-κB signaling pathway in hypothalamic microglia, which subsequently hyperactivates the HPA axis, thereby promoting thymic apoptosis and autophagy, impairing thymic development in neonatal rat, and ultimately contributing to systemic immunosuppression.

**Supplementary Information:**

The online version contains supplementary material available at 10.1186/s13578-026-01543-w.

## Introduce

Previously, most studies have focused on neuronal injury and neuroprotective strategies in neonatal hypoxic-ischemic encephalopathy (HIE). However, research on the systemic immune dysfunction induced by HIE remains limited. Growing evidence indicates that central nervous system (CNS) injury significantly influences the immune system in adults following cerebral ischemia [1, 2]. During the acute phase of stroke, immune organs become activated, releasing numerous of immune cells, cytokines and chemokines. These mediators induce the expression of adhesion molecules on vascular endothelial cells, facilitating immune cells infiltration into the brain and exacerbating neural damage [3, 4]. Within a few days, however, the damaged CNS exerts a potent immunosuppressive effect—manifested by lymphocyte loss in peripheral immune organs such as the spleen and thymus—and the bidirectional crosstalk between the CNS and the immune system collectively shapes post-ischemic brain injury outcomes [5]. Notably, children with HIE exhibit abnormal lymphocyte subsets distributions, which contribute to immune dysfunction and further aggravate brain injury [6]. These observations suggest that ischemic brain injury may impair the development of the neonatal immune system.

The thymus is a critical primary lymphoid organ that plays a central role in immune surveillance and homeostasis, defending against pathogen invasion and supporting the differentiation, development, and maturation of T lymphocytes [7]. Meanwhile, the hypothalamic-pituitary-adrenal (HPA) axis serves as a major neuroendocrine pathway through which the brain modulates immune function, regulating multiple stages of immune cell development and responsiveness [8]. The CNS can activate the HPA axis by upregulating corticotropin-releasing hormone (CRH) expression, leading to the release of glucocorticoids (GCs) that binding to glucocorticoid receptors (GR) on thymocytes and thereby influence thymocyte development and survival [9]. In adult animal models, cerebral ischemia activates the HPA axis, subsequently increasing adrenal cortex reactivity [10, 11]. Importantly, inhibition HPA axis activation in a middle cerebral artery occlusion (MCAO) mouse model reduces immune cell apoptosis, attenuates damage to peripheral immune organs, and alleviate systemic immunosuppression [12]. Together, these findings imply that cerebral ischemia may drive peripheral immunosuppression through HPA axis activation. Nevertheless, the precise mechanism underlying HPA axis activation in this context remains unclear.

Emerging evidence highlights that neuroinflammation—driven by activated microglia and their production of pro-inflammatory mediators—plays a pivotal role in neuronal injury in neonatal HIE [13–15]. Intriguingly, studies in adult models suggest that brain injury can activate the HPA axis thorough neuroinflammation signaling [12]. High mobility group box 1 (HMGB1) and Toll-like receptor 4 (TLR4) are key mediators in initiating and amplifying inflammatory and neuroinflammatory responses [16, 17]. However, it is unknown whether HMGB1/TLR4/NF-κB signaling-mediated neuroinflammation contributes to thymic dysfunction following neonatal cerebral ischemia.

Although the immune system acts as a natural checkpoint for inflammatory responses, both the CNS and immune system in neonates are still immature. Consequently, the interplay between brain injury and the immune regulation after hypoxia-ischemia may differ substantially from that observed in adults. Therefore, the present study investigates this relationship and the underlying mechanisms using a neonatal rat HIE model, with the aim of providing novel insights and a potential foundation for integrated therapeutic strategies targeting both central and peripheral compartments in children with HIE.

## Materials and experimental methods

### Animals

SPF-grade, Sprague Dawley (SD) adult rats were purchased from Hunan Slake Kingda Laboratory Animal Company Limited (License No. SCXK (Xiang) 2019-0004), with a mating ratio of 1 ♂: 2 ♀. Seven-day-old SD rats, weighing between 12 and 18 g, both male and female, were selected. All animals were housed at an ambient temperature of 22 ± 2 °C, relative humidity of 50 ± 5%, and a 12 h light/dark cycle, and were free to eat standard laboratory feed and water. All animal procedures and experimental protocols were approved by the Biomedical Research Ethics Committee of Gannan Medical University.

### Rat neonatal HIE modeling and antagonist administration

**Fig. 1:**
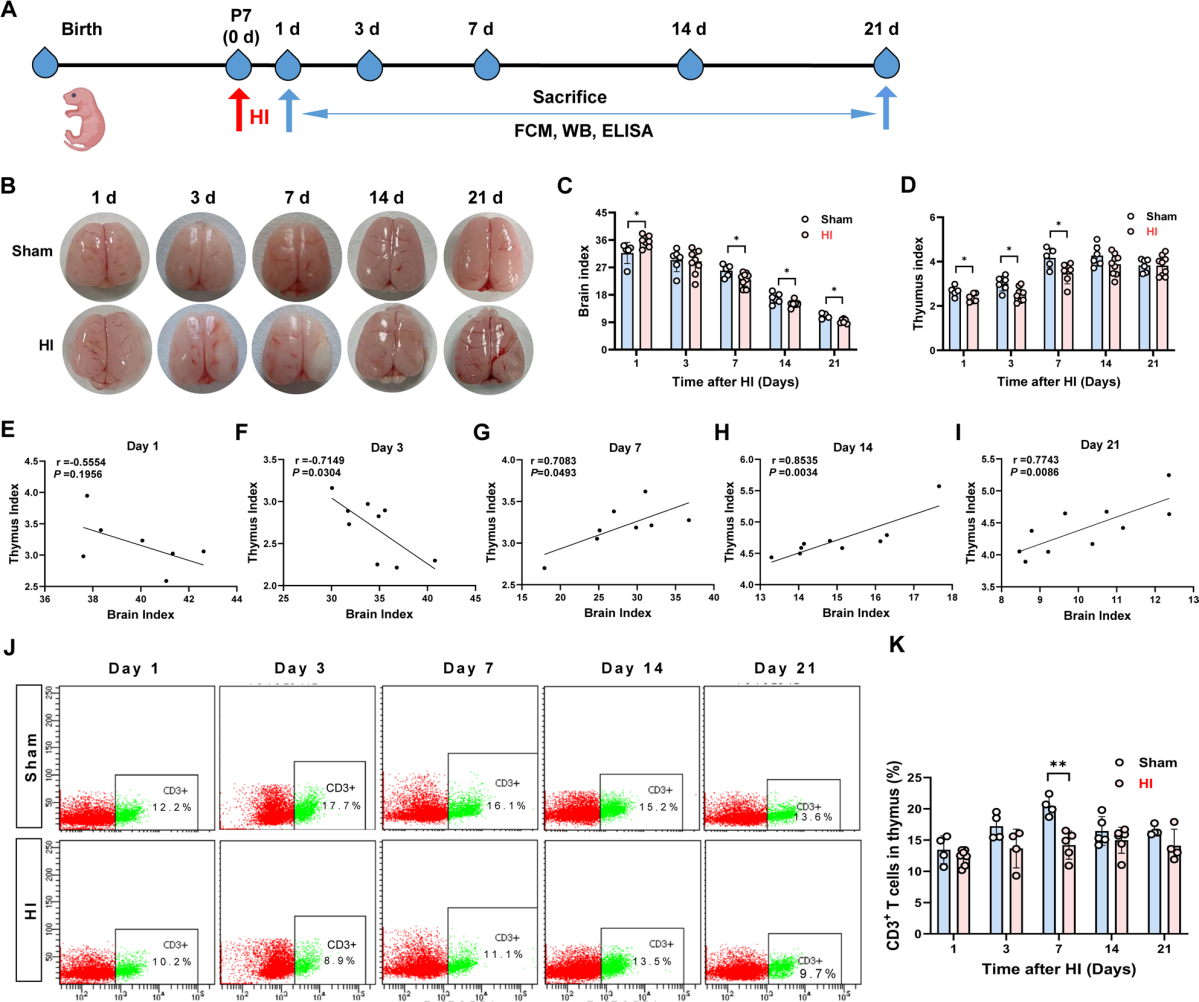
HI induced thymic atrophy and dysfunction in neonatal rats. The brain and thymus index, correlation analysis and thymic function were evaluated on days 1, 3, 7, 14 and 21 after HI. **A** Schematic diagram for experimental scheme. **B** Brain pictures. **C, D** Brain and thymus index, *n* = 7–10. **E–I** Correlation analysis of thymus index with brain index, *n* = 7–10. **J**–**K** Percentage of CD3^+^ T cell counts in thymus, *n* = 4–6. Data were expressed as the mean ± SD. Comparisons between the two groups were made using an unpaired T-test and Pearson’s test was used to analyze the correlation, ^*^P < 0.05, ^**^P < 0.01 versus Sham group.

The rat neonatal HIE model was established by Rice-Vannucci method [18]. After inhalation of isoflurane (Beikang, No. 21042801) (induced and maintained at a concentration of 2.0–3.0%) for anesthesia, the right common carotid artery was isolated and occluded using a unipolar electrocoagulation device, and finally sutured with tissue glue (3 M company, USA) (the whole process took less than 5 min). In Sham operation group, pups had only the right common carotid artery isolated without electrocoagulation. After regaining consciousness, the pups were immediately returned to the original mother’s cage for feeding. Newborn rats were allowed to recover for 1.5 h after ischemia, then placed in an oxygen dynamic detection box, with the temperature maintained at 38–39℃ and the relative humidity at 30–50%. The hypoxia concentration was set at 7.0% (filled with 93% N_2_), and the rats were treated with hypoxia for 1 h, then returned to the cage of the original mother rats for feeding after reoxygenation for 10 min (Fig. 1A).

To elucidate the effect of HMGB1 on the HPA axis and thymus, the HMGB1 antagonist GLY (DMSO formulated) (Selleck chem, USA) with a 20 mg/kg dose was injected intraperitoneally (at a concentration of 20 mg/mL) immediately after the HI and once daily thereafter. Equal volumes of the solvent were administered intraperitoneally to both the Sham and HI groups (Fig. 5A).To inhibit the HPA axis, the glucocorticoid receptor antagonist RU486 (MedChemExpress, USA) with a 10 mg/kg dose (DMSO: Corn Oil = 1:9) [8, 19] was administered intraperitoneally once a day (at a concentration of 6 mg/mL) at 12 h before and 2 h post-HI (Fig. 6A).

### Organ index

To evaluate the neonatal rat organ index post-HI, the thymus, whole brain, hypothalamus, pituitary gland and adrenal glands were harvested from neonatal rats on days 1, 3, 7, 14 and 21 post-HI surgery, weighed and recorded, and the corresponding organ index was calculated using the formula: Organ Index = Organ weight (mg) / Body weight (g).

### Flow cytometry

To detect thymocyte apoptosis and lymphocytes in neonatal rats post-HI, thymus samples collected on days 1, 3, 7, 14 and 21 post-HI were ground, filtered, and centrifuged. The supernatant was then discarded, and the pellet was resuspended with precooled PBS to prepare a single cell suspension (1–2 × 10^6^ cells/mL).

Apoptosis assay: A single-cell suspension at 1 × 10 ^5^ cells/mL was prepared in 1×Binding Buffer (BD, USA). 100 µL of the suspension was added to a flow cytometry tube, followed by the addition of Annexin V (BD, USA) and PI (BD, USA). Apoptosis was then measured and analyzed using a BD FACS Canto II flow cytometer.

Thymocyte count assay: 100 µL of the single-cell suspension was added into a flow cytometry tube, followed by the addition of PerCP/Cyanine 5.5 Anti-Rat CD3 Antibody (Elabscience, China). The sample was then analyzed using a BD FACS Canto II flow cytometer.

### ELISA (enzyme-linked immunosorbent assay)

Serum and hypothalamic tissues from neonatal rats were collected on days 1, 3, 7, 14, and 21 post-HI. Serum hormone levels, along with hypothalamic tissue hormone and cytokine levels, were measured using CRH, ACTH, and CORT ELISA kits (Omnimabs, USA), as well as TNF-α and IL-1β ELISA kits (Proteintech, China), following the manufacturers’ instructions.

### Western blot

Thymus and hypothalamus tissues were harvested from neonatal rats on days 1, 3, 7, 14, and 21 post-HI. Following the instructions of the BCA protein quantitation kit (Thermo, USA), the apoptosis-promoting proteins Cleaved-Caspase 3 (WL02117, Wanleibio), Bcl-2 (WL01556, Wanleibio) and Bax (WL01637, Wanleibio) in thymus were detected. Autophagic proteins Beclin-1 (66665-1, Proteintech), LC3 II/I (2775 S, CST), p-PI3K (ab302958, Abcam), PI3K (4257S, CST), p-Akt (WLP001a, Wanleibio), Akt (4060S, CST), p-mTOR (5536 S, CST), mTOR (2983, CST), GR (WL02695, Wanleibio), β-actin (sc-47778, Santa Cruz) and Histone H3 (4499, CST) in thymus were detected. Additionally, HMGB1 (ab18256, Abcam), TLR4 (sc-293072, Santa Cruz), p-P65-NF-κB (AN371-1, Beyotime), P65-NF-κB (WL01980, Wanleibio), TNF-α (ab6671, Abcam) and CD11b (ab133357, Abcam) in hypothalamus were detected. Imaging was performed using the Amersham Imager 600 instrument (USA), and the bands were captured and analyzed with ImageJ software.

### Cytoplasmic protein and nucleoprotein extraction

Neonatal rat thymus tissues were harvested on the day 7 post-HI. The extraction of thymus tissue plasma and nuclear proteins was conducted according to the instructions of the Nuclear Plasma Protein Extraction Kit (Wanleibio, China), and the samples were used to detect thymus cytoplasmic and intranuclear GR expression levels.

### Statistical analysis

The results of this experiment were statistically analyzed using GraphPad Prism 10.1.2 software (USA). An Unpaired T-test was used for comparisons between two groups, one-way ANOVA was employed to analyze differences among multiple groups followed by Dunnett’s test, and the Pearson correlation coefficient was utilized to assess the linear relationship between two variables. All experimental data are expressed as mean ± standard deviation (Mean ± SD), and the results were considered statistically significant at *P* < 0.05.

## Results

### HIE induced thymic atrophy and thymic dysfunction in neonatal rats

Thymus and brain tissue samples were collected from rats on days 1, 3, 7, 14, and 21 post-HI (Fig. 1A). As illustrated in Fig. 1B, neonatal rats in the HI group exhibited significant brain edema on the first day compared with the Sham group, with some reduction by the third day, followed by brain atrophy and liquefaction on day 7, 14 and 21. The brain index exhibited a significantly increase on day 1 post-HI, indicative of acute cerebral edema. By day 3, the brain index did not differ significantly from that of the Sham group. From day 7 onward, the brain index progressively decreased, becoming significantly lower than the Sham group on days 14 and 21, suggesting chronic brain atrophy (Fig. 1C). The thymus index showed a significant reduction on days 1, 3, and 7 post-HI, but remained unchanged on days 14 and 21 (Fig. 1D). A negative correlation between thymus and brain indices was observed on days 1 and 3 post-HI (Fig. 1E–F), whereas a positive correlation was noted on days 7, 14, and 21 (Fig. 1G–I). These results indicate that brain injury is accompanied by thymus atrophy, suggesting a potential mutual regulatory mechanism between the two.

The thymus serves as the primary site for T lymphocyte proliferation, differentiation, and maturation. CD3^+^ is a critical surface marker indicative of the immune response. Compared with the Sham group, the percentage of CD3^+^ T lymphocytes in the thymus of HI group rats did not change significantly on days 1, 3, 14 and 21, but decreased on the day 7 (Fig. 1J–K).

### HIE induced apoptosis in neonatal rat thymocytes

**Fig. 2:**
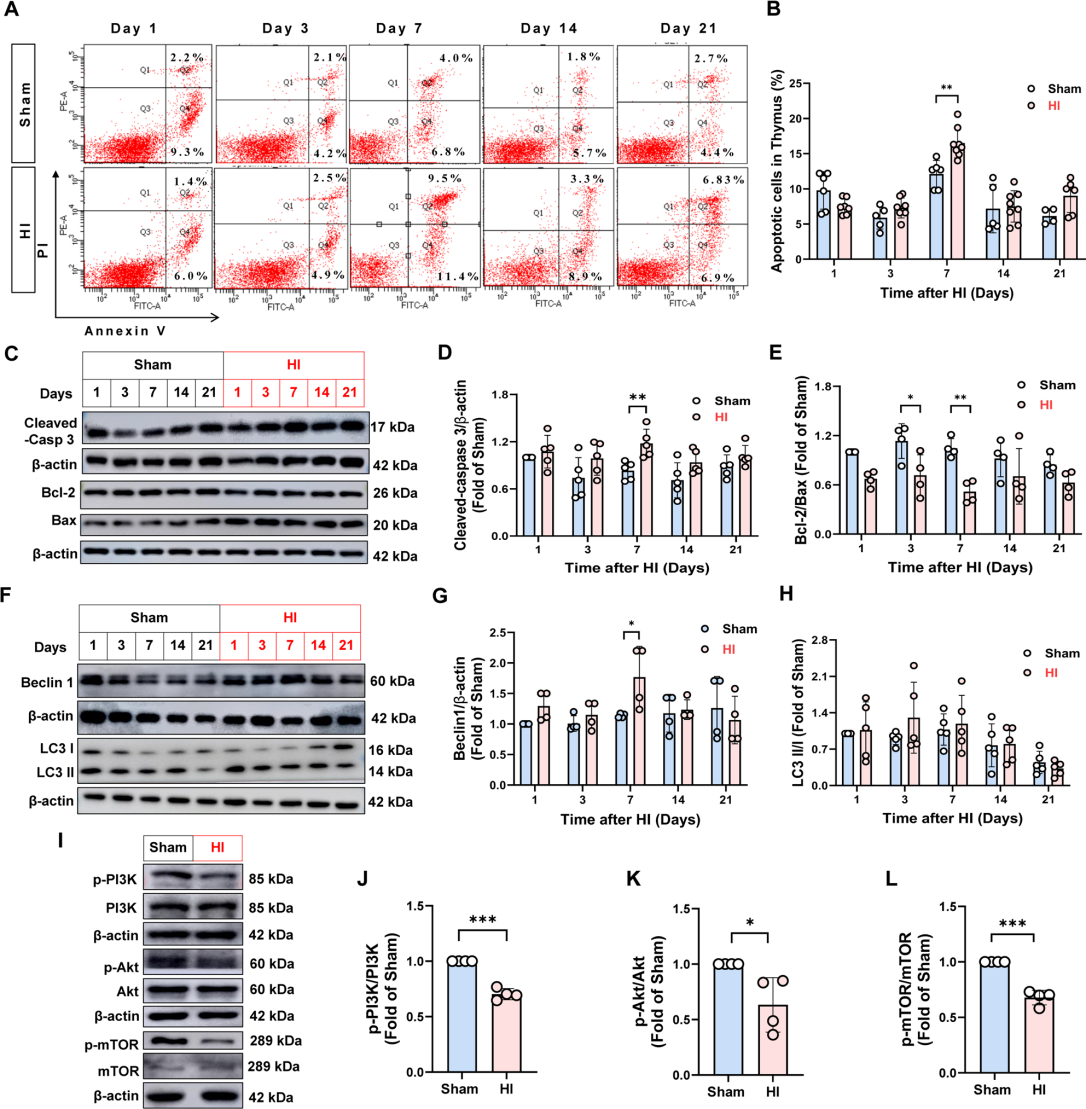
HI induced thymocyte apoptosis and autophagy in neonatal rats.The thymocyte apoptosis, autophagy and the activations of PI3K/Akt/mTOR signaling in thymus were evaluated on days 1, 3, 7, 14 and 21 after HI. **A**–**B** Percentage of thymocyte apoptosis, *n* = 5–8. **C**–**E** Protein levels of cleaved-caspase 3, Bcl-2/Bax in thymus, *n* = 4–5. **F**–**H** Beclin-1 and LC3 II/I protein expressions, *n* = 4–5. **I**–**L** p-PI3K, p-Akt, and p-mTOR protein expressions, *n* = 4. Data were expressed as the mean ± SD. Comparisons between the two groups were made using an unpaired T-test, ^*^P < 0.05, ^**^P < 0.01, ^***^P < 0.001 versus Sham group.

It’s well-established that Annexin V^+^/PI^−^ cells represents early apoptotic cell, while Annexin V^+^/PI^+^ indicate late apoptotic cells. As shown in the results of Fig. 2A–B, the percentage of apoptotic thymocytes in the HI group tend to increase on days 3, 7, 14 and 21 post-HI, with the most pronounced elevation observed on day 7 (*P* < 0.05). Correlation analysis between the percentage of thymocyte apoptosis and brain index (Supplementary Fig. 1A-E), revealed a positive correlation on days 1 and 3, and a negative correlation on days 7, 14 and 21, suggesting that brain injury may trigger thymocyte apoptosis. Additionally, correlation analysis between thymocyte apoptosis and thymus index (Supplementary Fig. 1F-J) showed a negative correlation on days 1 and 3, and a positive correlation on days 7, 14, and 21, indicating that increased thymocyte apoptosis contributes to the reduction in thymus index, during the chronic phase. Furthermore, Western blot analysis (Fig. 2C–E) revealed a significant upregulation in the expression levels of cleaved-caspase 3 in the HI group on day 7 (*P* < 0.05), indicating heightened apoptotic activity. Simultaneously, the ratios of Bcl-2/Bax was significantly decreased in the HI group on days 3 and 7 (*P* < 0.05), reflecting a shift toward pro-apoptotic signaling. Together, these findings suggested that HI-induced thymic atrophy is closely associated with increased thymocyte apoptosis, mediated by dysregulation of key apoptotic pathways including caspase 3 activation and Bcl-2/Bax imbalance.

### HIE induced autophagy in neonatal rat thymocytes

It is well known that the relationship between apoptosis and autophagy is complex and dynamic, involving mutual regulation through cooperative, antagonistic, or reciprocal interactions. Western blot analysis of thymic tissues various time points revealed dynamic changes in both apoptotic and autophagy-related proteins following HI (Fig. 2F–H). In comparison to the Sham group, the HI group showed a progressive increase in Beclin-1 expression from day 1 to day 7, with peak levels observed on day 7. Based on this temporal profile, day 7 was identified as the critical time point for further mechanistic exploration.

The PI3K/AKT/mTOR signaling pathway is a pivotal intracellular pathway regulating metabolism, inflammation, cell growth and survival [20], and functions as a major suppressor of autophagy under physiological conditions. To investigate its role in thymic autophagy after HI, we investigated the phosphorylation status of PI3K, Akt, and mTOR in the thymus on day 7 post-HI. As shown in Fig. 2I–L, phosphorylation levels of PI3K, Akt, and mTOR were significantly reduced in the HI group compared with the Sham group (*P* < 0.01), indicating inhibition of the PI3K/Akt/mTOR signaling pathway. This suggests that reduced PI3K/Akt/mTOR signaling, particularly decreased mTOR phosphorylation, may contribute to HI-induced autophagy in neonatal thymocytes.

### HIE induced HPA axis activation in neonatal rat

**Fig. 3:**
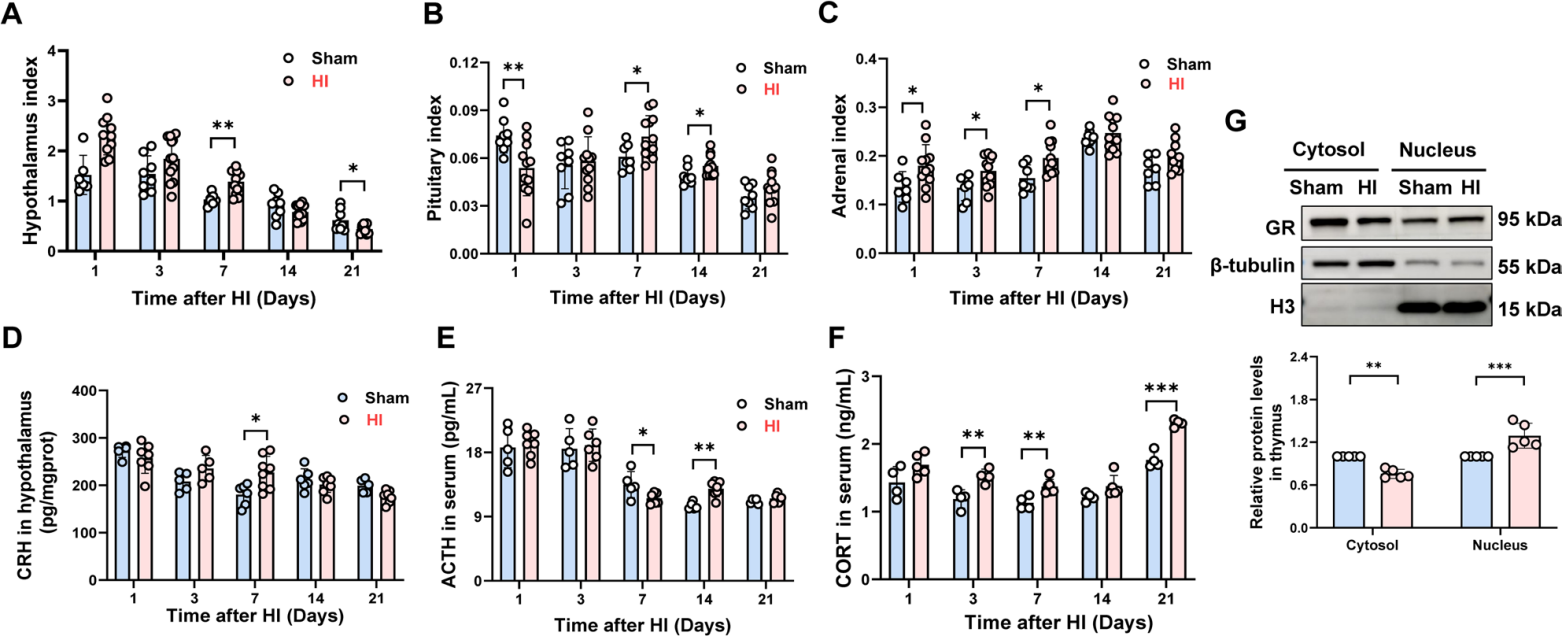
HIE induced HPA axis activation in neonatal rats.The HPA axis activation were evaluated on days 1, 3, 7, 14 and 21 after HI, and the protein levels of GR in thymus were detected on the day 7 after HI. **A**–**C** hypothalamic, pituitary, and adrenal index, *n* = 7–12. **D** CRH content in hypothalamus, *n* = 5–8. **E**–**F** ACTH and CORT content in serum, *n* = 4–8. **G** Intracytoplasmic and intranuclear GR in thymus, *n* = 5. Data were expressed as the mean ± SD. Comparisons between the two groups were made using an unpaired T-test, *P < 0.05, **P < 0.01, ***P < 0.001 vs. Sham group

Precise regulation of HPA axis activity is essential for maintaining organismal homeostasis, as overactivation leads to excessive release GCs into the circulation, resulting in severe metabolic and immune disturbances [8]. Given the critical bidirectional communication between the CNS and the immune system, the brain can modulate immune cell development and all stages of the immune response via the HPA axis—both systems being vulnerable to ischemia-reperfusion injury [21]. Therefore, we explored functional alterations in the HPA axis following HI. The hypothalamic index increased significantly on day 7 post-HI, and then tended to recover (Fig. 3A). The pituitary index decreased significantly on the day 1, but increased significantly on days 7 and 14 (Fig. 3B). The adrenal index increased significantly on days 1, 3 and 7 (Fig. 3C). Hypothalamic CRH levels showed no significant chang on day 1 post-HI, exhibited an increasing trend on day 3, increased significantly on the day 7 (*P* < 0.05), and then tended to decrease thereafter (Fig. 3D). Serum ACTH levels did not change significantly from the Sham group on day 1 and 3, decreased significantly on day 7 (*P* < 0.05), rose markedly on day 14 (*P* < 0.01), and returned to baseline by day 21 (Fig. 3E). In contrast, Serum CORT levels increased significantly on days 3, 7 and 21 (*P* < 0.05), with a non-significant increasing trend observed on days 1 and 14 (Fig. 3F). Furthermore, HI induced significant GR nuclear translocation in thymocytes, reflected by reduced cytoplasmic and elevated nuclear GR levels (*P* < 0.01) (Fig. 3G). These findings indicate that HI activates the HPA axis—evidenced by elevated CRH and CORT—and enhances GR nuclear translocation in thymocytes.

### HIE caused hypothalamic microglial activation and neuroinflammation

**Fig. 4:**
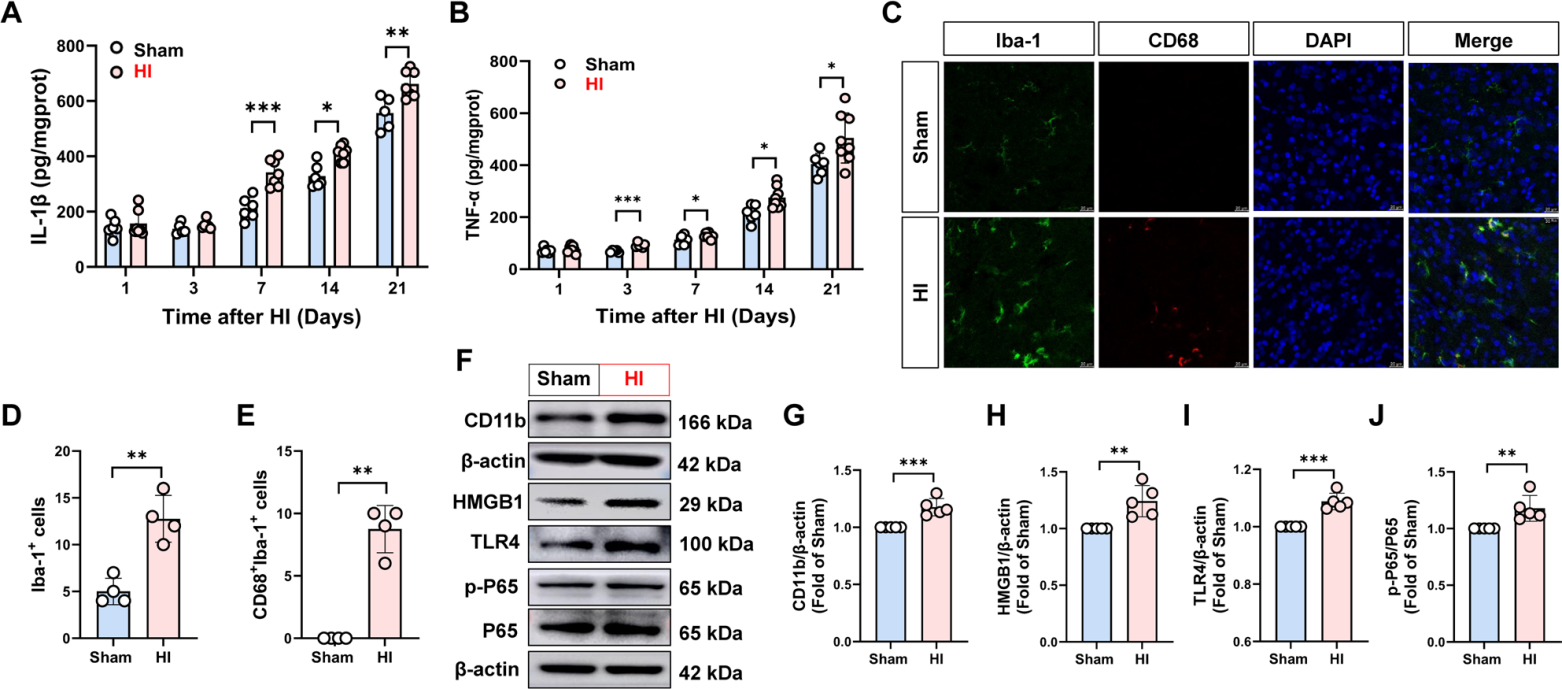
HI induced the activations of TLR4/HMGB1/NF-κB signaling and microglia, leading to neuroinflammation in hypothalamus. **A**–**B **TNF-α and IL-1β on days 1, 3, 7, 14 and 21 after HI, *n* = 5–8. **C** Double immunofluorescence staining of Iba-1 and CD68. Scale bar = 20 μm. **D** Number of Iba-1 positive cells (*n* = 4). **E** CD68^+^/ Iba-1^+^ cells rate (*n* = 4). **F**–**J** CD11b, HMGB1, TLR4, p-P65-NF-κB and P65-NF-κB protein levels on day 7 after HI, *n* = 5. Data were expressed as the mean ± SD. Comparisons between the two groups were made using an unpaired T-test, ^*^P < 0.05, ^**^P < 0.01, ^***^P < 0.001 versus Sham group.

It is well established that proinflammatory cytokines such as TNF-α, IL-1β and IL-6 can activate CRH neurons in the hypothalamus, thereby stimulating HPA axis activity [22]. To investigate the mechanisms underlying hypothalamic activation following HI in neonatal rats, we assessed the levels of key inflammatory mediators. As shown in Fig. 4A–B, hypothalamic IL-1β level showed no significant change on days 1 and 3 post-HI but were significantly elevated on days 7, 14, and 21 (*P* < 0.05). Similarly, TNF-α expression remained unchanged on day 1 but increased significantly from day 3 onward (*P* < 0.05), suggesting that HI induces sustained neuroinflammatory responses in the hypothalamus. Given that TNF-α, IL-1β, and IL-6 are primarily released by activated microglia, and that hypothalamic microglia can sense external stimuli—including GCs and infection—to modulate neuroendocrine function [23], we further examined microglial responses after HI. Immunofluorescence analysis revealed that microglia in the HI group adopted a typical activated morphology—characterized by enlarged cell bodies, shortened and thickened processes, and reduced branching—whereas microglia in the Sham group exhibited a resting morphology with slender processes and a dense network (Fig. 4C). Quantitatively, Iba-1fluresences intensity was significantly higher in the HI group (*P* < 0.001) (Fig. 4C, D). Moreover, CD68-positive cells—indicative of phagocytic, pro-inflammatory M1-polarized microglia—were absent in the Sham group but markedly present in the HI group (*P* < 0.001) (Fig. 4C–E). Consistent with this, the protein level of the microglial marker CD11b in the hypothalamus of the HI group was significantly increased (*P* < 0.001) (Fig. 4F, G). These data indicated that HI triggered microglial activation in the hypothalamus, leading to the release of significant amounts of cytokines, which may influence HPA axis activity.

As an important inflammatory mediator that initiates the neuroinflammatory responses, HMGB1 can regulate CORT-induced microglial activation and cytokine secretion. Therefore, we further examined whether the HMGB1/TLR4/NF-κB pathway contributes to microglial activation post-HI. Compared with the Sham group, the hypothalamic levels of HMGB1, TLR4, and phosphorylated NF-κB proteins were significantly increased in the HI group (*P* < 0.01) (Fig. 4F, H-J). Collectively, these results suggest that HI may promote microglia-mediated neuroinflammation by activating the HMGB1/TLR4/NF-κB signaling pathway, potentially contributing to HPA axis hyperactivity and thymocyte apoptosis.

### Inhibition of HMGB1 alleviated HIE-induced dysregulation of the HPA axis and thymic injury

**Fig. 5:**
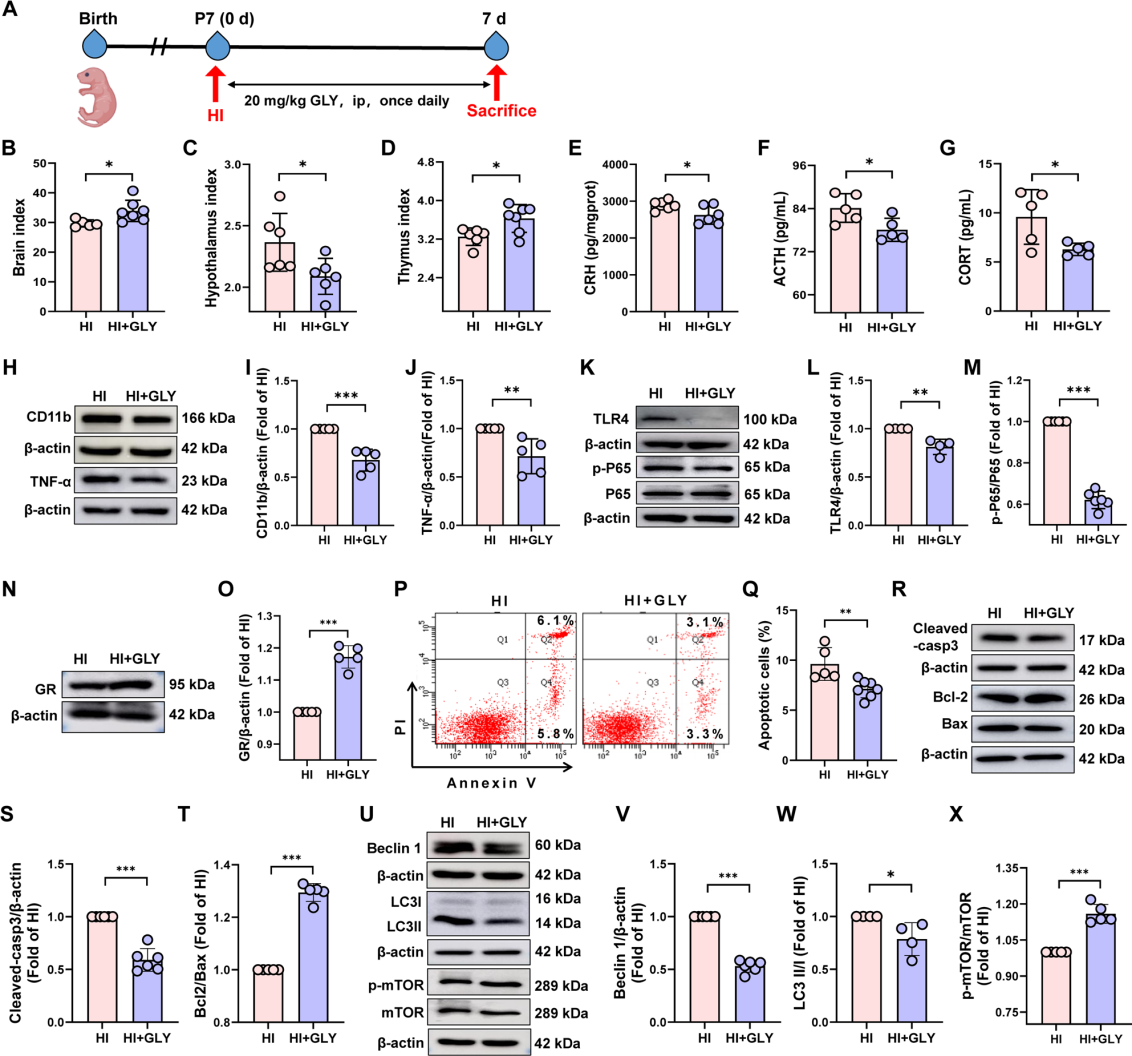
GLY alleviated the activation of HPA axis and thymus disorders in neonatal HI rats.The neonatal rats were treated with GLY (a HMGB1 inhibitor), and the samples were obtained on day 7 after HI to evaluate the activation of HPA axis and thymus disorders. **A** Schematic diagram for experimental scheme. **B**–**D** Brain, hypothalamus and thymus index, *n* = 5–7. **E** CRH content in hypothalamus, *n* = 6. **F**–**G** ACTH and CORT content in serum, *n* = 5. **H**–**M** CD11b, TNF-α, TLR4, p-P65-NF-κB and P65-NF-κB protein levels in hypothalamus, *n* = 4–6. **N**–**O** GR protein level in thymocytes, *n* = 5. **P–Q** Percentage of thymocyte apoptosis, *n* = 5–7. **R–X** Cleaved-caspase 3, Bcl-2/Bax, Beclin-1, LC3 II/I and p-mTOR/mTOR protein levels in thymus, *n* = 5–6. Data were expressed as the mean ± SD. Comparisons between the two groups were made using an unpaired T-test, ^*^P < 0.05, ^**^P < 0.01, ^***^P < 0.001 versus HI group.

To further clarify the role of HMGB1 in HI-induced HPA axis activation, we used the HMGB1 inhibitor GLY to suppress HMGB1 signaling in the hypothalamus and assessed its effects on the HPA axis and thymus (Fig. 5A). Compared with HI-injured neonatal rats, GLY treatment significantly increased the brain index (*P* < 0.05) (Fig. 5B), and decreased the hypothalamic index (Fig. 5C). It also markedly decreased hypothalamic levels of CRH, as well as serum ACTH and CORT (*P* < 0.05) (Fig. 5E–G). Concurrently, GLY significantly reduced CD11b, TNF-α, TLR4 and p-P65-NF-κB protein levels in hypothalamus (*P* < 0.01) (Fig. 5H–J, K–M), indicating attenuation of microglial activation and neuroinflammation. In the thymus, GLY restored the expression levels of GR protein (*P* < 0.001) (Fig. 5N–O) and normalized the thymus-to-body weight ratio (*P* < 0.05) (Fig. 5D). It also reduced thymocyte apoptosis (*P* < 0.01) (Fig. 5P–Q), downregulated pro-apoptotic proteins Cleaved-caspase 3 and Bax, and markedly upregulated the anti-apoptotic protein Bcl-2 and the Bcl-2/Bax ratio (*P* < 0.001) (Fig. 5R–T), collectively alleviating thymic atrophy. Furthermore, GLY significantly reduced the expression of autophagy-related proteins Beclin-1 and LC3 II/I (*P* < 0.001), and enhanced mTOR phosphorylation (*P* < 0.001) (Fig. 5U–X), suggesting restoration of autophagic homeostasis in the thymus.

### Glucocorticoid receptor antagonism alleviated HIE-induced HPA axis dysregulating and thymic injury

**Fig. 6:**
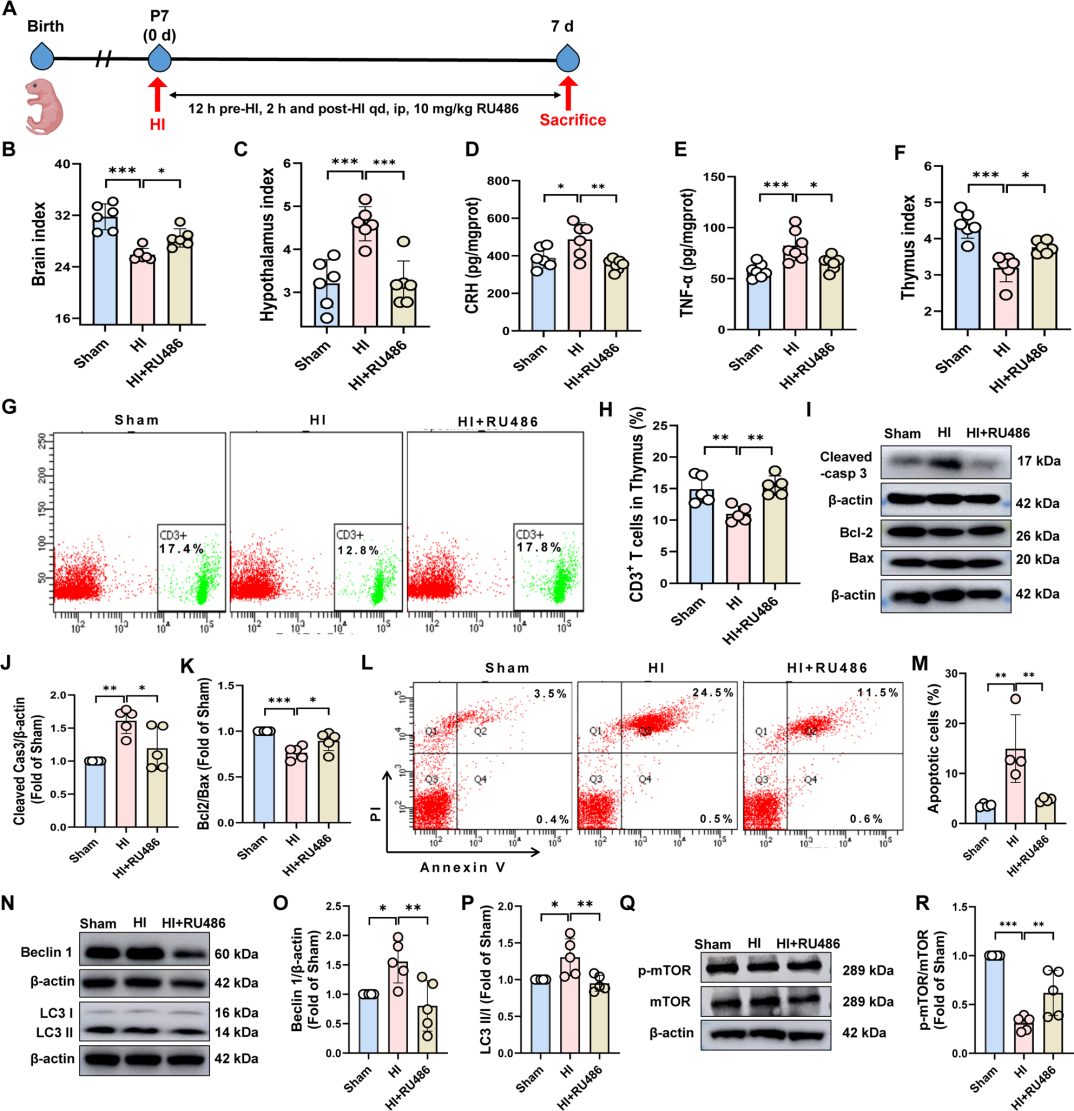
RU486 relieved the effect of HIE on HPA axis and thymus.The neonatal rats were treated with RU486 (a GR inhibitor), and the samples were obtained on day 7 after HI to evaluate the activation of HPA axis and thymus disorders. **A** Schematic diagram for experimental scheme. **B**–**C** Brain and hypothalamic index, *n* = 6. **D**–**E** CRH and TNF-α content in hypothalamus, *n* = 6–7. **F** Thymic index, *n* = 6. **G**–**H** Percentage of CD3^+^ T lymphocytes in thymus, *n* = 5. **I**–**K** Cleaved-caspase 3, Bcl-2/Bax protein levels in thymus, *n* = 5. **L**–**M** Percentage of thymocyte apoptosis, *n* = 4. **N**–**R** Beclin-1, LC3 II/I and p-mTOR/mTOR protein levels in thymus, *n* = 5. Data were expressed as the mean ± SD. Comparisons between groups were performed using one-way ANOVA followed by Dunnett’s test. ^*^P < 0.05, ^**^P < 0.01, ^***^P < 0.001 versus HI group.

To further investigate whether glucocorticoid signaling via HPA axis activation contributes to HI-induced apoptosis and autophagy in neonatal murine thymocytes, we used glucocorticoid receptor antagonist RU486 to inhibit GR signaling (Fig. 6A). As illustrated in Fig. 6B, RU486 significantly increased the brain index (*P* < 0.05), while concurrently decreasing the hypothalamic index and downregulating hypothalamic expression of CRH and TNF-α (*P* < 0.05) (Fig. 6C–E). These effects collectively inhibited the activation of the HPA axis and ameliorated brain damage. Additionally, RU486 also restored the thymus index (*P* < 0.05) (Fig. 6F) and the density of CD3^+^ T-cell (Fig. 6G–H). It also significantly decreased the percentage of apoptosis thymocytes (*P* < 0.01) (Fig. 6L–M), suppressed pro-apoptotic proteins Cleaved-caspase 3 and Bax, and markedly upregulated the anti-apoptotic proteins Bcl-2 and Bcl-2/Bax ratio (*P* < 0.01) (Fig. 6I–K), collectively alleviating thymic atrophy. Furthermore, RU486 significantly reduced the autophagy-related proteins Beclin-1 and LC3 II/I (*P* < 0.01) (Fig. 6N–P) and enhanced phosphorylation of mTOR (*P* < 0.01) (Fig. 6Q–R), suggesting restoration of autophagic homeostasis in thymocytes.

## Discussion

In this study, we confirmed that cerebral HI induces both apoptosis and autophagy in neonatal rat thymocytes, disrupts thymic development, and leads release proinflammatory factors via the HMGB1/TLR4/NF-κB signaling pathway, thereby exacerbating HPA axis overactivation and modulating thymocyte apoptosis and autophagy.

The thymus is the primary site of T-cell maturation. During late gestation and the neonatal period, mature T lymphocytes begin to migrate from the thymus to periphery lymphoid organs [24]. Infancy represents a critical window of thymic expansion, during which its volume and function are highly susceptible to external factors such as malnutrition and disease. The neonatal thymus plays a crucial role in establishing the body’s immune system. Its removal triggers rapid atrophy of lymphoid organs, lymphopenia, and impaired immune responsiveness, leading to an increased risk of infections, premature immunosenescence, malignancies, and autoimmune disorders [25, 26]. Atrophy of immune organs and lymphopenia have been observed in experimental stroke models [27]. In this study, we found that the brain post-HI exhibited cerebral edema in the acute phase and cerebral liquefaction and infarction in the chronic phase, accompanied by thymic atrophy. This atrophy exhibited a negative correlation with brain-to-body weight ratios in the early phase (1–3 days). That is, the more severe the brain injury was, the more pronounced the cerebral edema became, and the higher the brain-to-body weight ratio was. Moreover, it was positively correlated with the brain-to-body weight ratios in the chronic phase, which was consistent with hypoxia-induced thymus gland degeneration during childhood [28]. In addition, the reduction in thymus volume in neonatal rats post-HI was accompanied by a significant decrease in the number of CD3^+^ T lymphocytes, likely due to impaired thymic function affected T-lymphocyte homing and egress. This finding is consistent with results reported in the ischemia-reperfusion injury model of adult SD rats [29, 30]. Collectively, these data suggest that HI contributes to thymic atrophy and subsequent immunosuppression.

Apoptosis and autophagy serve as essential regulatory measures of cellular homeostasis and fate. Under physiological conditions, senescent or damaged cells are eliminated via apoptosis [31, 32]. The intrinsic (mitochondrial) and extrinsic (death receptor) pathways converge on the activation of effector caspases, notably cleaved caspase-3, which is recognized as the hallmark executioner of apoptosis [33]. Bcl-2 (anti-apoptotic) and Bax (pro-apoptotic) critically regulate mitochondrial outer membrane permeabilization, thereby controlling caspase activation. Autophagy—a lysosome-dependent catabolic process classified as type II programmed cell death—maintains cellular homeostasis by degrading misfolded proteins and damaged organelles [31, 32]. While basal autophagy is cytoprotective, excessive or dysregulated autophagy under pathological conditions (e.g., ischemia/hypoxia) can promote cell death. This process is tightly regulated by the PI3K/Akt/mTOR pathway, wherein mTOR acts as a key negative regulator of autophagy initiation [33, 34]. Crosstalk between apoptosis and autophagy has been documented in various pathologies, including excessive ethanol exposure [35] and viral infections [36], both of which can induce thymic apoptosis and autophagosome formation. In this study, we found that in the thymus of neonatal rats post-HI, protein levels of cleaved caspase-3 and Bax were progressively increased, while Bcl-2 was decreased in a time-dependent manner—indicating that thymocyte apoptosis intensified with ongoing thymic injury. Concurrently, autophagy-related protein levels (e.g., LC3II, Beclin-1) increased over time, peaking on day 7, coinciding with reduced phosphorylation of key components in the PI3K/Akt/mTOR signaling pathway. These correlative observations suggest that suppression of this pathway may be associated with enhanced autophagic activity following HI. We therefore hypothesize that inhibition of mTOR—potentially downstream of PI3K/Akt—may relieve its tonic suppression of autophagy, thereby facilitating autophagosome formation. However, it should be noted that this proposed regulatory mechanism remains inferential and has not yet been functionally validated. Future inhibitor-based studies will be essential to establish a causal link between PI3K/Akt/mTOR signaling and HI-induced autophagy in the neonatal thymus. Collectively, these findings suggest that HI-induced thymocyte apoptosis and excessive autophagy may be key contributors to thymic atrophy.

The CNS communicates with the immune system via three pathways: the HPA axis, the sympathetic nervous system, and the parasympathetic nervous system—with the HPA axis serving as the dominant neuroendocrine conduit [37]. The hypothalamus, pituitary, and adrenal glands form a tightly regulated negative feedback loop. Hypothalamic CRH neurons in paraventricular nucleus (PVN) secrete CRH into the hypophyseal portal system, stimulating pituitary ACTH release and subsequent adrenal GC production, which initiates a negative feedback loop in the HPA axis by activating GR in the brain to suppress its own synthesis [37]. CRH, ACTH, and GC exert bidirectional effects on lymphocyte proliferation: low concentrations promote, while high concentrations inhibit immune cell activity [38]. In adult ischemic stroke, hyperactivity of the HPA axis triggers the CRH → ACTH → GC cascade, resulting in GR-mediated apoptosis of immune cells and systemic immunosuppression [9, 12]. Correspondingly, HI neonates exhibited elevated hypothalamic CRH and serum CORT levels, along with enlargement of the hypothalamus and adrenal glands on days 1, 3, and 7 post-HI. Thereafter hypothalamic volume and CRH secretion normalized. In contrast, the adrenal glands remained enlarged and continued to secrete GC, likely due to high GC levels exerting negative feedback on hypothalamic CRH secretion and attenuating HPA-axis reactivity. Conversely, the GR antagonist RU486 inhibited the activation of the HPA axis. We also observed that during the acute phase of HI, pituitary feedback inhibition was reduced and ACTH secretion declined; however, both the pituitary index and ACTH secretion subsequently increased significantly—the mechanism underlying this biphasic response remains unclear.

Microglia, the resident immune cells of the CNS, are swiftly activated by ischemic insults, leading to the release of proinflammatory cytokines (e.g., TNF-α, IL-1β), amplifying neuroinflammation and neuronal injury [39]. The HMGB1/TLR4/NF-κB pathway is a well-established mediator of sterile inflammation [40]. Normally localized in the nucleus, HMGB1 translocates to the cytoplasm and is actively secreted or passively released upon cellular stress (e.g., HI), acting as a damage-associated molecular pattern (DAMP) to activate TLR4 on microglia [41–44]. TLR4 is predominantly expressed in microglia and promotes their activation, leading to inflammation and dysfunction of hypothalamic PVN [45]. Activated microglia can induce GC production via the HPA axis, which in turn further activates microglia, generating a vicious cycle [46–48]. In adult mice with ischemic stroke, GCs bind to GR on microglia and induce HMGB1 production and release, which—through TLR4 activation—upregulates the expression of inflammatory mediators such as NF-κB, TNF-α, and IL-1β, ultimately exacerbating neuroinflammation [47, 49, 50]. Following administration of GR antagonists, the expression of HMGB1 and inflammatory mediators was significantly reduced [47, 51]. Similarly, in this study, we found that microglial activation, HMGB1/TLR4/NF-κB pathway activity, and proinflammatory cytokine release were all increased in the hypothalamus of newborn rats with HIE.

To investigate whether thymic dysfunction originates from neuroinflammation-induced overactivation of the HPA axis, we administered the HMGB1 inhibitor GLY and GR antagonist RU486. Both interventions suppressed the HMGB1/TLR4/NF-κB pathway, reduced microglia activation and cytokine release, attenuated HPA axis hyperactivity, and restored thymic GR expression and function. These results suggest the existence of a feed-forward loop: HI leads to hypothalamic microglial activation via the HMGB1/TLR4/NF-κB pathway, resulting in HPA axis overactivation and a subsequent glucocorticoid (GC) surge. This surge induces GR-mediated thymocyte apoptosis and autophagy, culminating in thymic atrophy and immunosuppression. Furthermore, elevated GCs may further promote microglial activation, thereby helping to sustain the cycle.

**Fig. 7:**
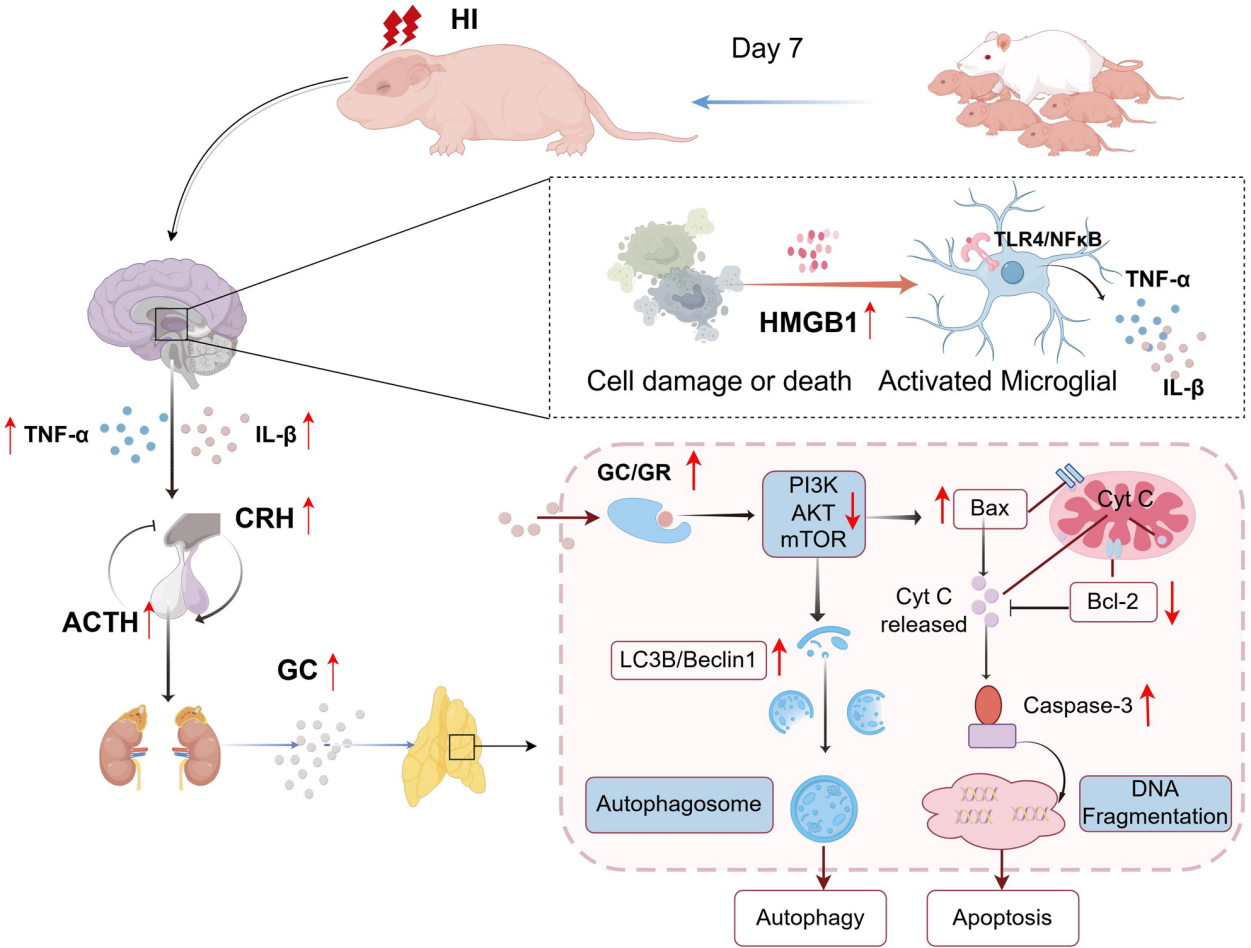
Schematic diagram of the impact of hypoxic-ischemic encephalopathy on neonatal immunity. HIE may precipitate neuroinflammation through the activation of HMGB1/TLR4/NF-κB signaling pathway in hypothalamic microglia, which subsequently activates the HPA axis to induce apoptosis and autophagy, thereby affecting thymic development in neonatal rat and culminating in systemic immunosuppression.

In summary, HI triggers hypothalamic microglial activation through the HMGB1/TLR4/NF-κB pathway, leading to excessive production of pro-inflammatory cytokines, HPA axis hyperactivity, and dysregulation of thymocyte apoptosis and autophagy—partly via suppression of the PI3K/Akt/mTOR pathway—culminating in thymic atrophy and immunosuppression (Fig. 7). In turn, high concentrations of GC may act on hypothalamic microglia to further enhance their activation, contributing to a vicious cycle of HPA axis overactivation. Thus, modulating HPA axis overactivation may serve as a critical target for enhancing immunity in HIE neonates and improving the efficacy of rehabilitation therapy.

## Supplementary Information

Below is the link to the electronic supplementary material.

Supplementary Figure 1. Brain index, thymocyte apoptosis and thymus index in neonatal rats are closely related. The correlation analysis was evaluated on the day 1, 3, 7, 14 and 21 after HI. (A–E) The correlation between the percentage of thymocyte apoptosis and brain index, n=5–7. (H–L) The correlation between the percentage of thymocyte apoptosis and thymus index, n=5–8. (F–J) Protein levels of cleaved-caspase 3, Bcl-2/Bax in thymus, n=4–5. Pearson's test was used to analyze the correlation.

Supplementary Material 2

## Data Availability

The data supporting the results of this study are available from the corresponding authors according to reasonable requirements.

## Abbreviations

HIE: Hypoxia–ischemic encephalopathy
MCAO: Middle cerebral artery occlusion
HPA: Hypothalamic–pituitary–adrenal axis
HI: Hypoxic-ischemic
CNS: Central nervous system
CRH: Corticotropin-releasing hormone
ACTH: Adrenocorticotropic hormone
GC: Glucocorticoid
CORT: Cortisol
GR: Glucocorticoid receptor
HMGB1: High mobility group box 1
TLR4: Toll-like receptor 4
NF-κB: Nuclear factor kappa B
SD: Sprague Dawley
RU486: Mifepristone
GLY: Glycyrrhizin
ELISA: Enzyme-linked immunosorbent assay
TNF-α: Tumor necrosis factorα
IL-1β: Interleukin-1β
ANOVA: Analysis of variance
