# Supplementary figures and images for: The mechanisms by which hypothalamic neuroinflammation induced by neonatal cerebral ischemia–hypoxia leads to decreased thymic function via the HPA axis
Source: Cell Biosci. 2026 Feb 23;16:38. doi: 10.1186/s13578-026-01543-w (PMC13036901; doi:10.1186/s13578-026-01543-w)

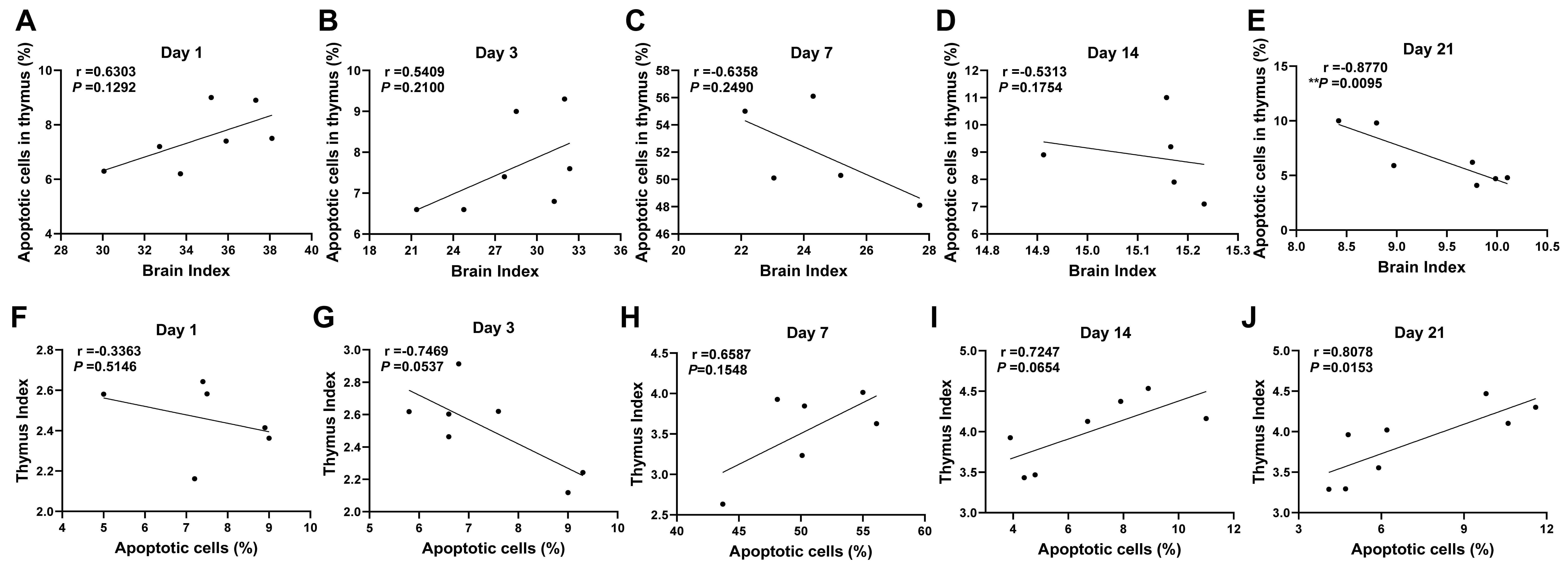

Supplement: Supplementary file 1 — Supplementary Figure 1. Brain index, thymocyte apoptosis and thymus index in neonatal rats are closely related. The correlation analysis was evaluated on the day 1, 3, 7, 14 and 21 after HI. (A–E) The correlation between the percentage of thymocyte apoptosis and brain index, n=5–7. (H–L) The correlation between the percentage of thymocyte apoptosis and thymus index, n=5–8. (F–J) Protein levels of cleaved-caspase 3, Bcl-2/Bax in thymus, n=4–5. Pearson's test was used to analyze the correlation. [file 13578_2026_1543_MOESM1_ESM.tif]
