# Supplementary material for: The mechanisms by which hypothalamic neuroinflammation induced by neonatal cerebral ischemia–hypoxia leads to decreased thymic function via the HPA axis
Source: Cell Biosci. 2026 Feb 23;16:38. doi: 10.1186/s13578-026-01543-w (PMC13036901; doi:10.1186/s13578-026-01543-w)
Supplement: Supplementary file 2 — Supplementary Material 2 [file 13578_2026_1543_MOESM2_ESM.pptx]

## Slide 1
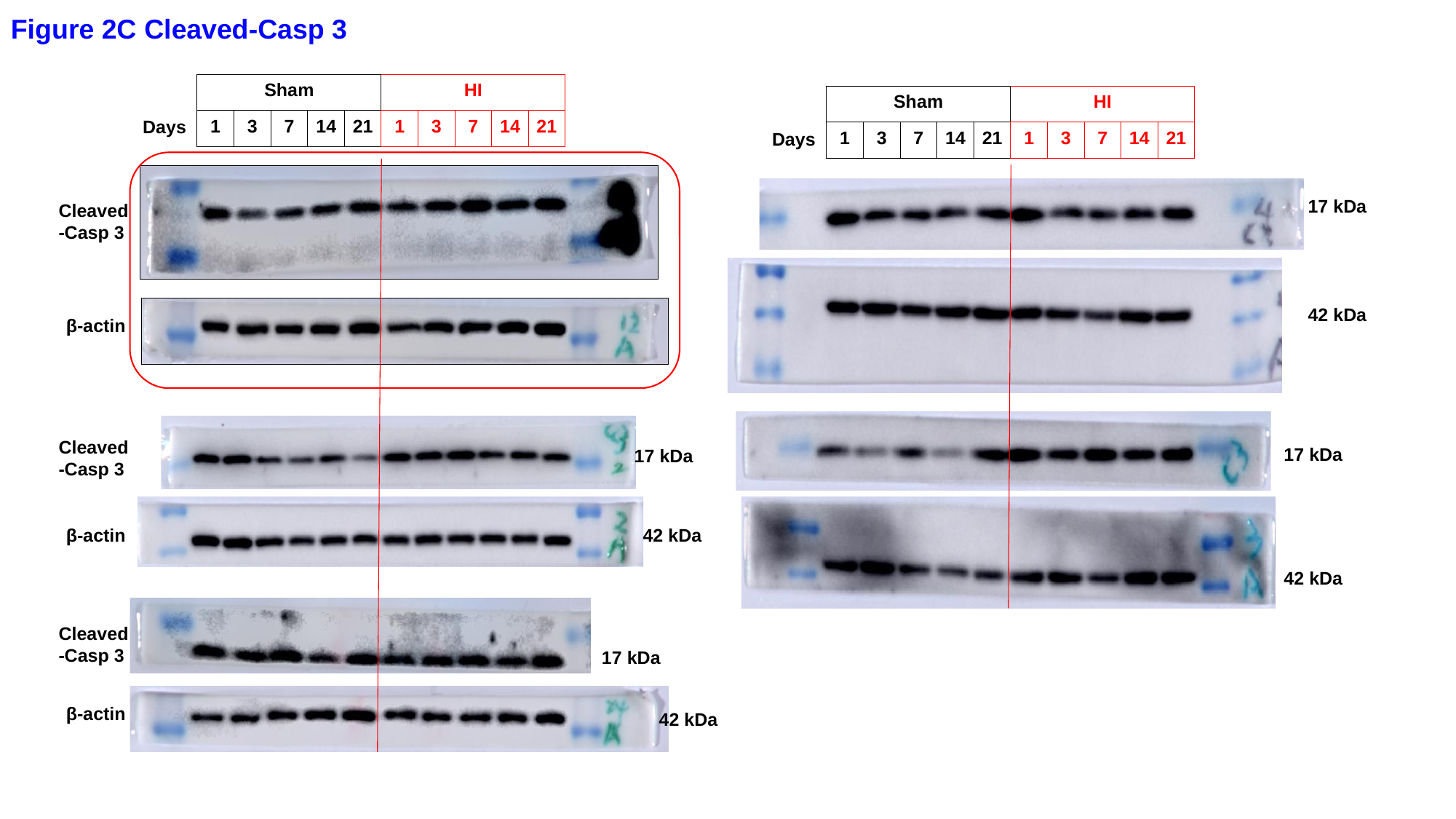

Figure 2C Cleaved-Casp 3
| Sham | | | | | HI | | | | |
| --- | --- | --- | --- | --- | --- | --- | --- | --- | --- |
| 1 | 3 | 7 | 14 | 21 | 1 | 3 | 7 | 14 | 21 |
| Sham | | | | | HI | | | | |
| --- | --- | --- | --- | --- | --- | --- | --- | --- | --- |
| 1 | 3 | 7 | 14 | 21 | 1 | 3 | 7 | 14 | 21 |
Days
Days
17 kDa
Cleaved-Casp 3
42 kDa
β-actin
Cleaved-Casp 3
17 kDa
17 kDa
β-actin
42 kDa
42 kDa
Cleaved-Casp 3
17 kDa
β-actin
42 kDa
1

## Slide 2
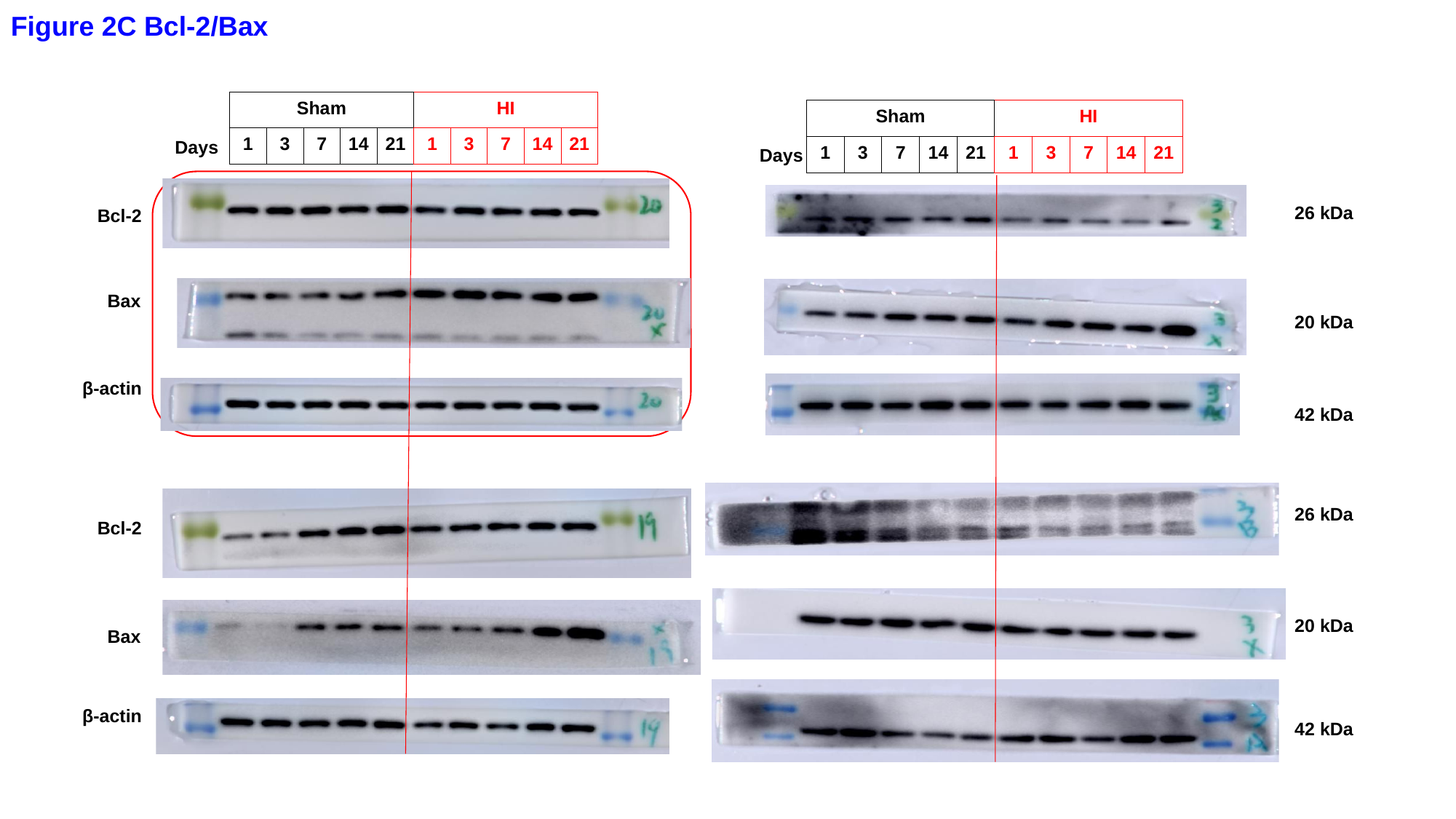

Figure 2C Bcl-2/Bax
| Sham | | | | | HI | | | | |
| --- | --- | --- | --- | --- | --- | --- | --- | --- | --- |
| 1 | 3 | 7 | 14 | 21 | 1 | 3 | 7 | 14 | 21 |
| Sham | | | | | HI | | | | |
| --- | --- | --- | --- | --- | --- | --- | --- | --- | --- |
| 1 | 3 | 7 | 14 | 21 | 1 | 3 | 7 | 14 | 21 |
Days
Days
26 kDa
Bcl-2
Bax
20 kDa
β-actin
42 kDa
26 kDa
Bcl-2
20 kDa
Bax
β-actin
42 kDa
2

## Slide 3
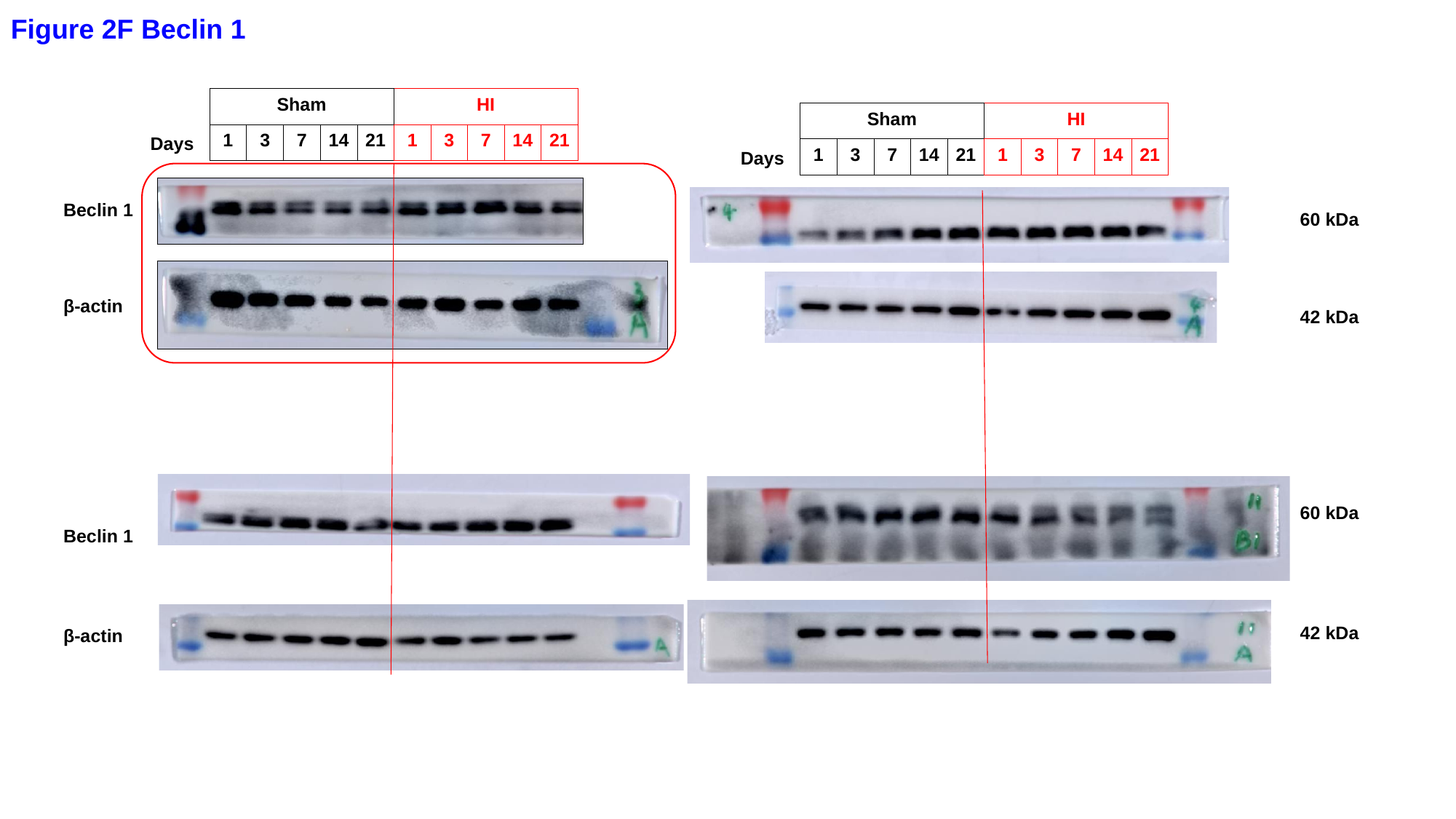

Figure 2F Beclin 1
| Sham | | | | | HI | | | | |
| --- | --- | --- | --- | --- | --- | --- | --- | --- | --- |
| 1 | 3 | 7 | 14 | 21 | 1 | 3 | 7 | 14 | 21 |
| Sham | | | | | HI | | | | |
| --- | --- | --- | --- | --- | --- | --- | --- | --- | --- |
| 1 | 3 | 7 | 14 | 21 | 1 | 3 | 7 | 14 | 21 |
Days
Days
Beclin 1
60 kDa
β-actin
42 kDa
60 kDa
Beclin 1
42 kDa
β-actin

## Slide 4
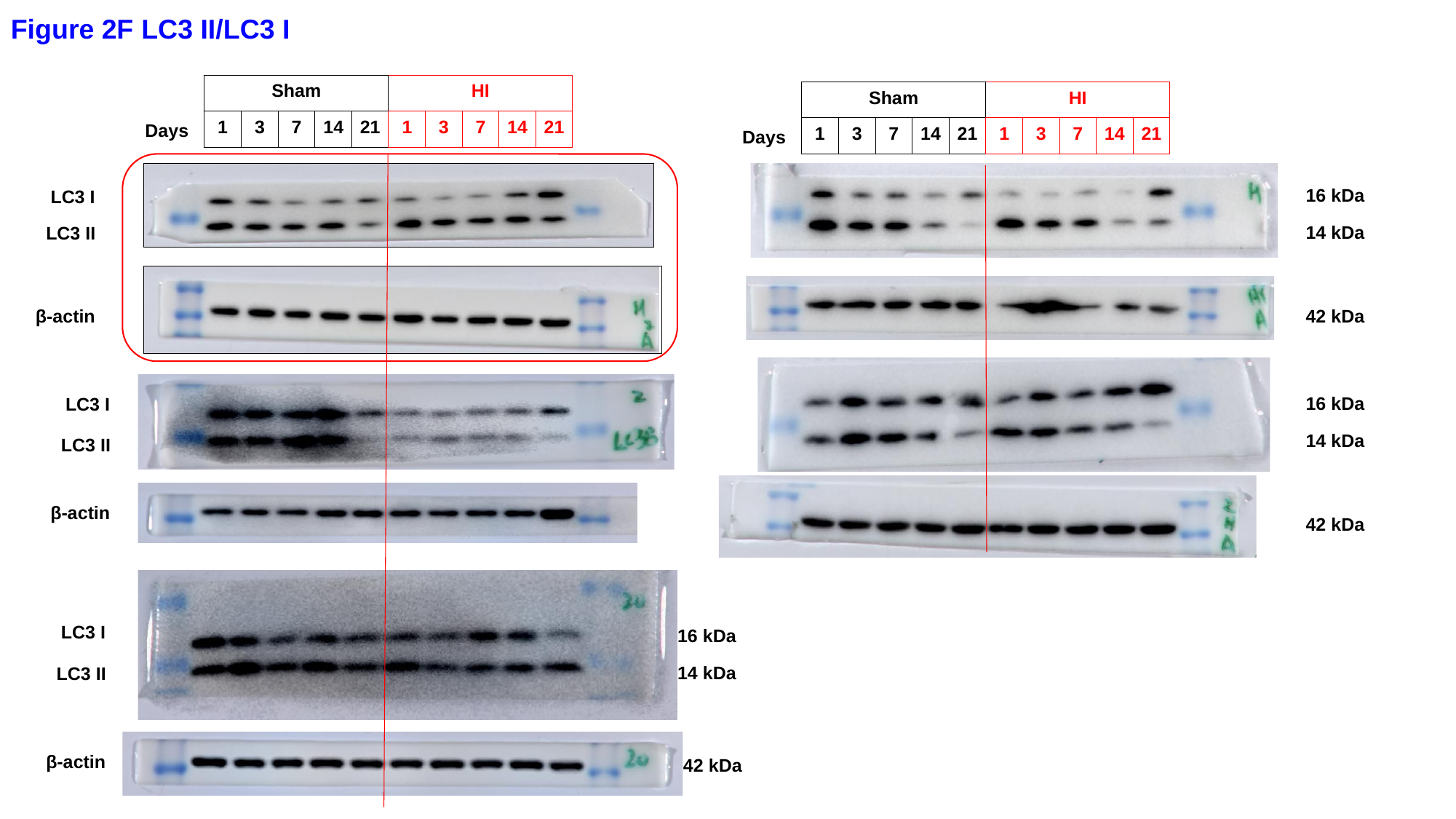

Figure 2F LC3 II/LC3 I
| Sham | | | | | HI | | | | |
| --- | --- | --- | --- | --- | --- | --- | --- | --- | --- |
| 1 | 3 | 7 | 14 | 21 | 1 | 3 | 7 | 14 | 21 |
| Sham | | | | | HI | | | | |
| --- | --- | --- | --- | --- | --- | --- | --- | --- | --- |
| 1 | 3 | 7 | 14 | 21 | 1 | 3 | 7 | 14 | 21 |
Days
Days
16 kDa
LC3 I
14 kDa
LC3 II
42 kDa
β-actin
16 kDa
LC3 I
14 kDa
LC3 II
β-actin
42 kDa
LC3 I
16 kDa
14 kDa
LC3 II
β-actin
42 kDa

## Slide 5
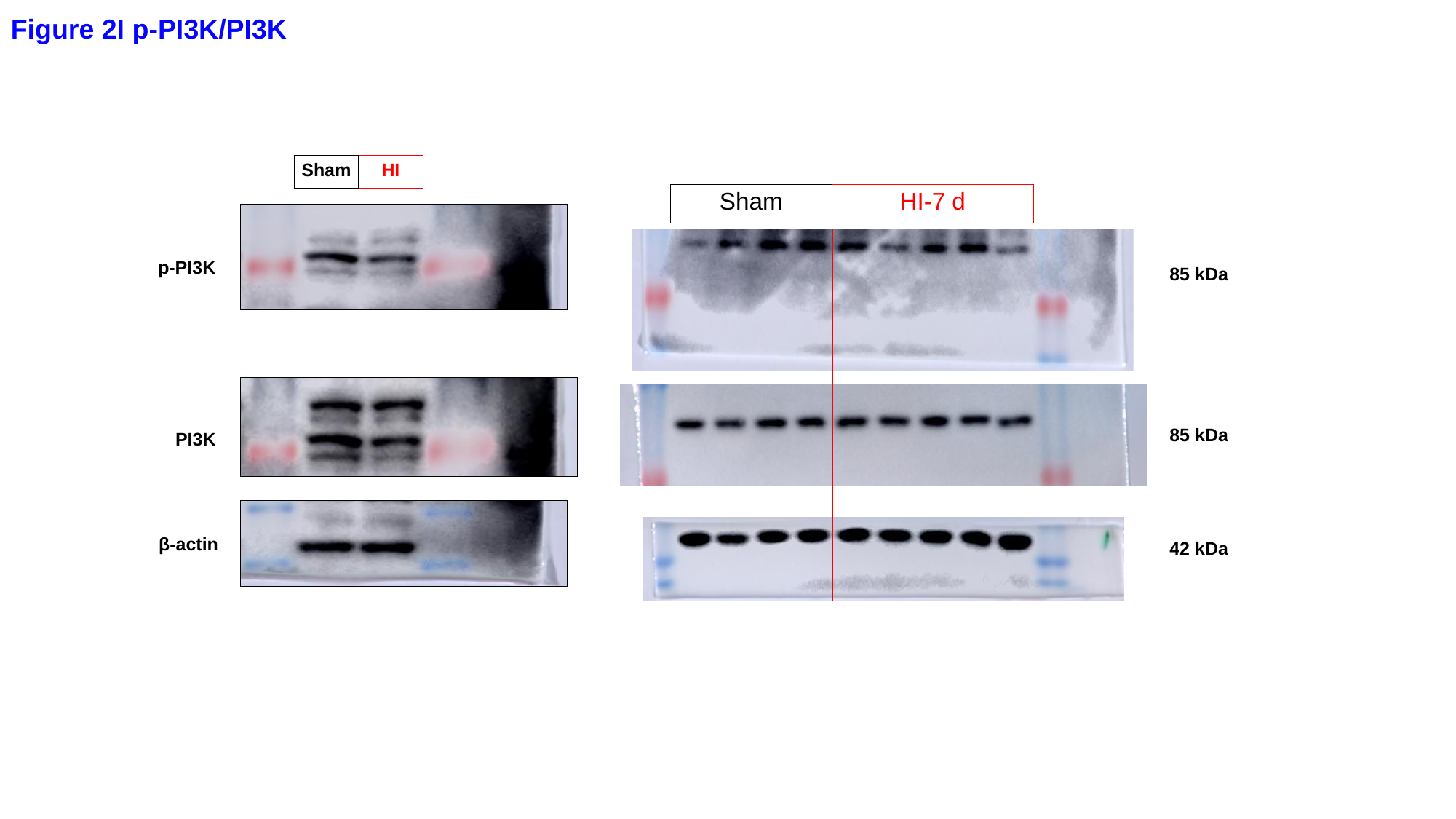

Figure 2I p-PI3K/PI3K
| Sham | HI |
| --- | --- |
| Sham | HI-7 d |
| --- | --- |
p-PI3K
85 kDa
85 kDa
PI3K
β-actin
42 kDa

## Slide 6
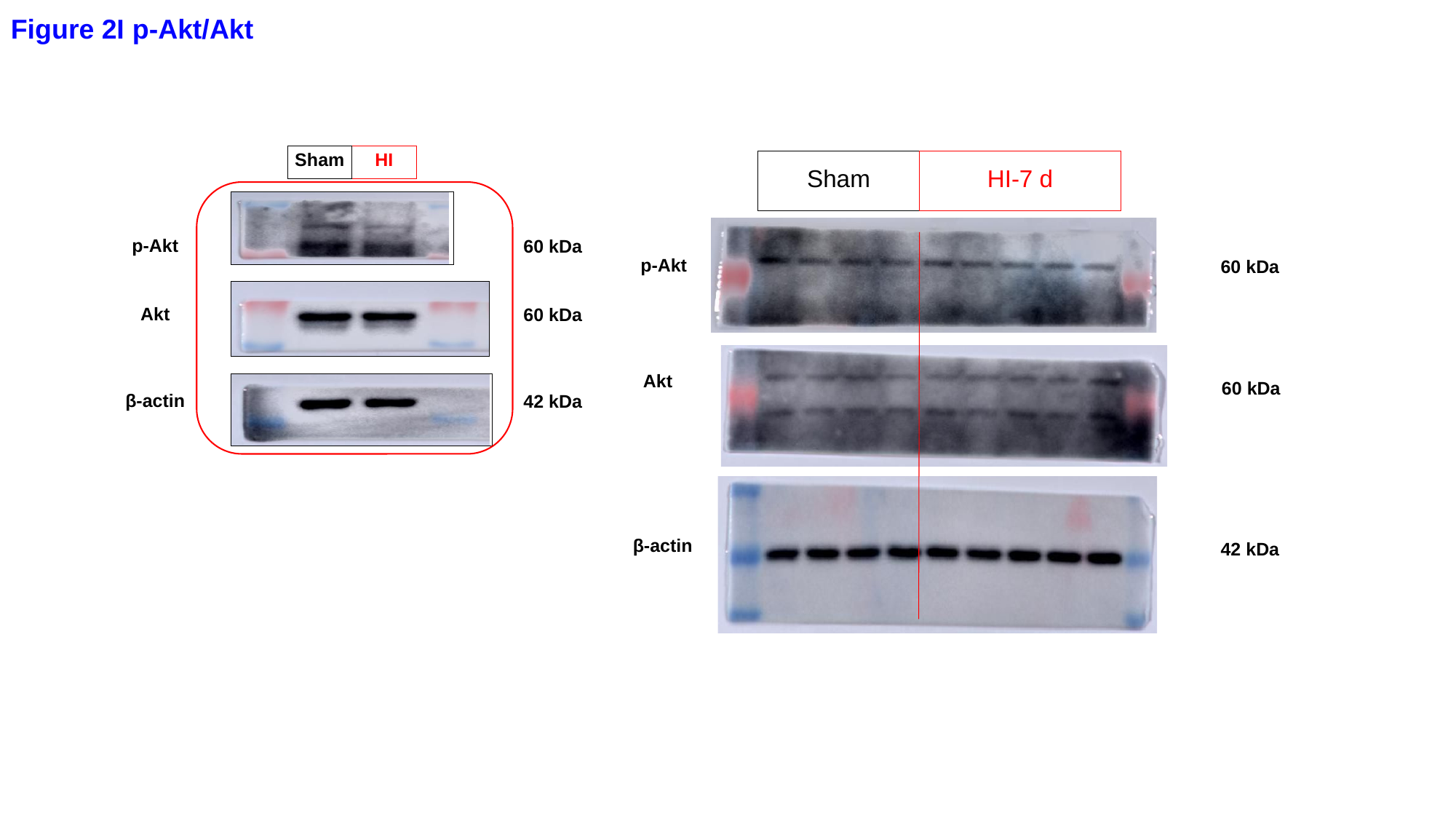

Figure 2I p-Akt/Akt
| Sham | HI |
| --- | --- |
| Sham | HI-7 d |
| --- | --- |
p-Akt
60 kDa
p-Akt
60 kDa
Akt
60 kDa
Akt
60 kDa
β-actin
42 kDa
β-actin
42 kDa

## Slide 7
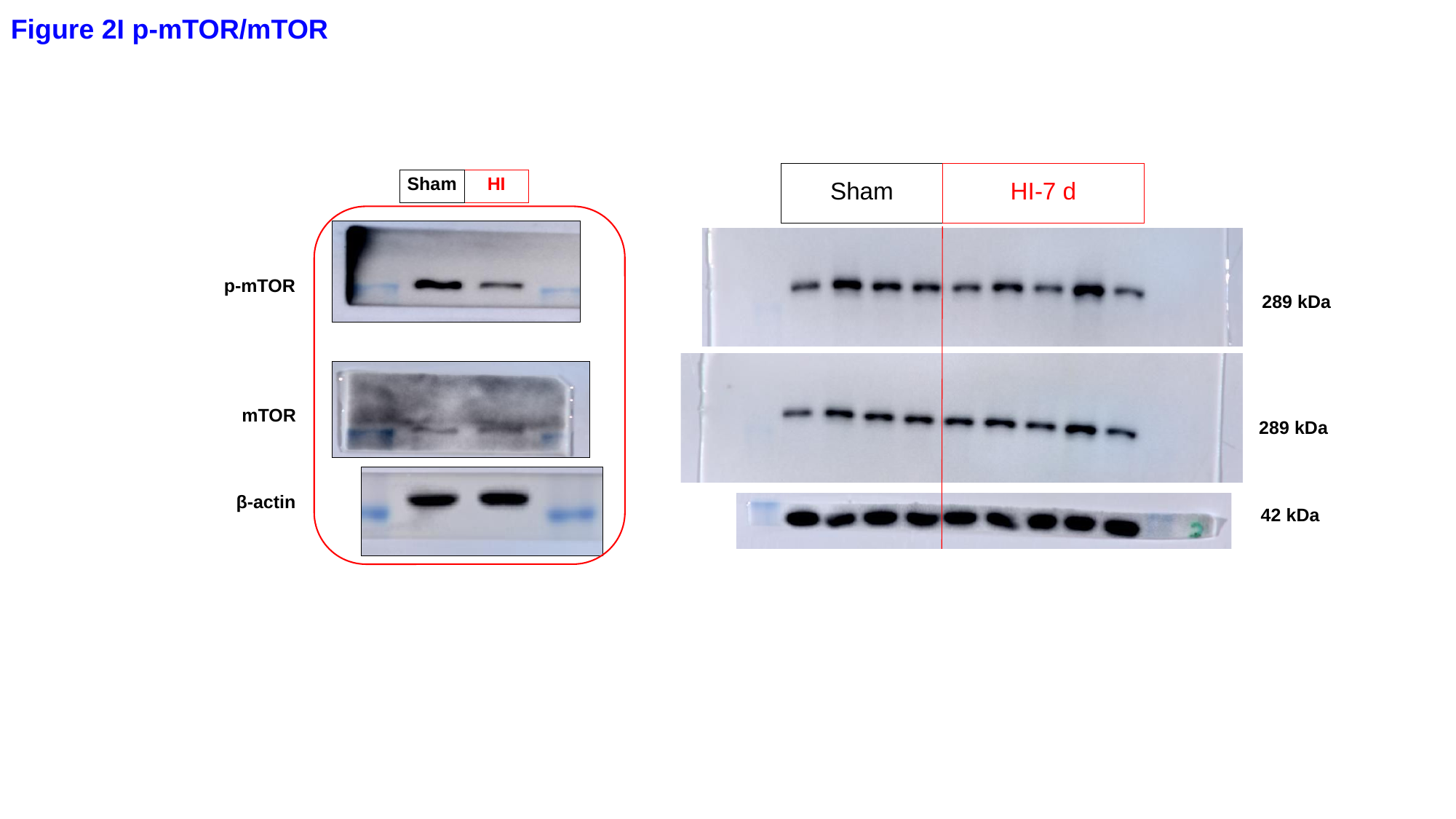

Figure 2I p-mTOR/mTOR
| Sham | HI-7 d |
| --- | --- |
| Sham | HI |
| --- | --- |
p-mTOR
289 kDa
mTOR
289 kDa
β-actin
42 kDa

## Slide 8
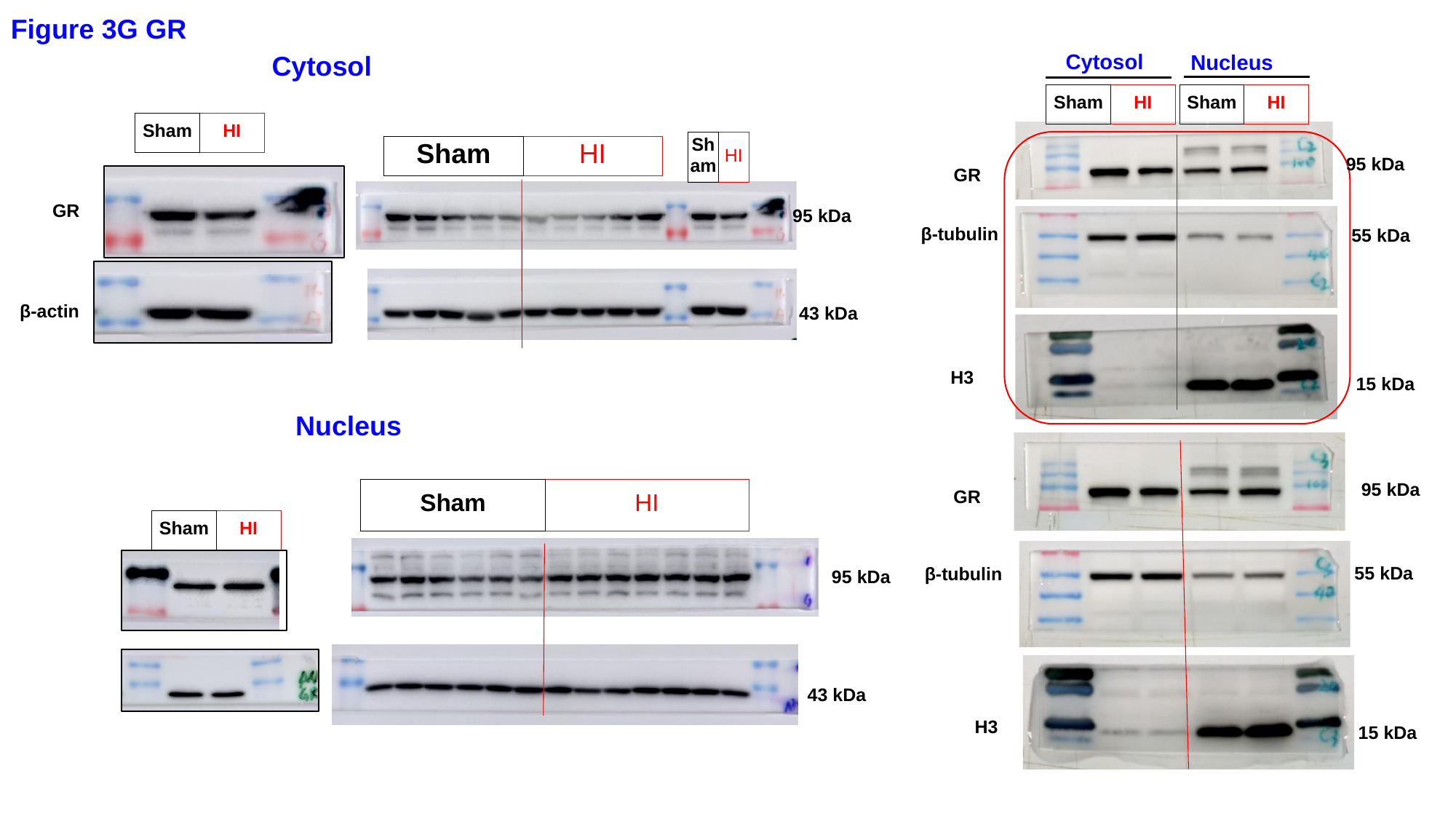

Figure 3G GR
Cytosol
Cytosol
Nucleus
| Sham | HI |
| --- | --- |
| Sham | HI |
| --- | --- |
| Sham | HI |
| --- | --- |
| Sham | HI |
| --- | --- |
| Sham | HI |
| --- | --- |
95 kDa
GR
GR
95 kDa
β-tubulin
55 kDa
β-actin
43 kDa
H3
15 kDa
Nucleus
95 kDa
| Sham | HI |
| --- | --- |
GR
| Sham | HI |
| --- | --- |
55 kDa
β-tubulin
95 kDa
43 kDa
H3
15 kDa

## Slide 9
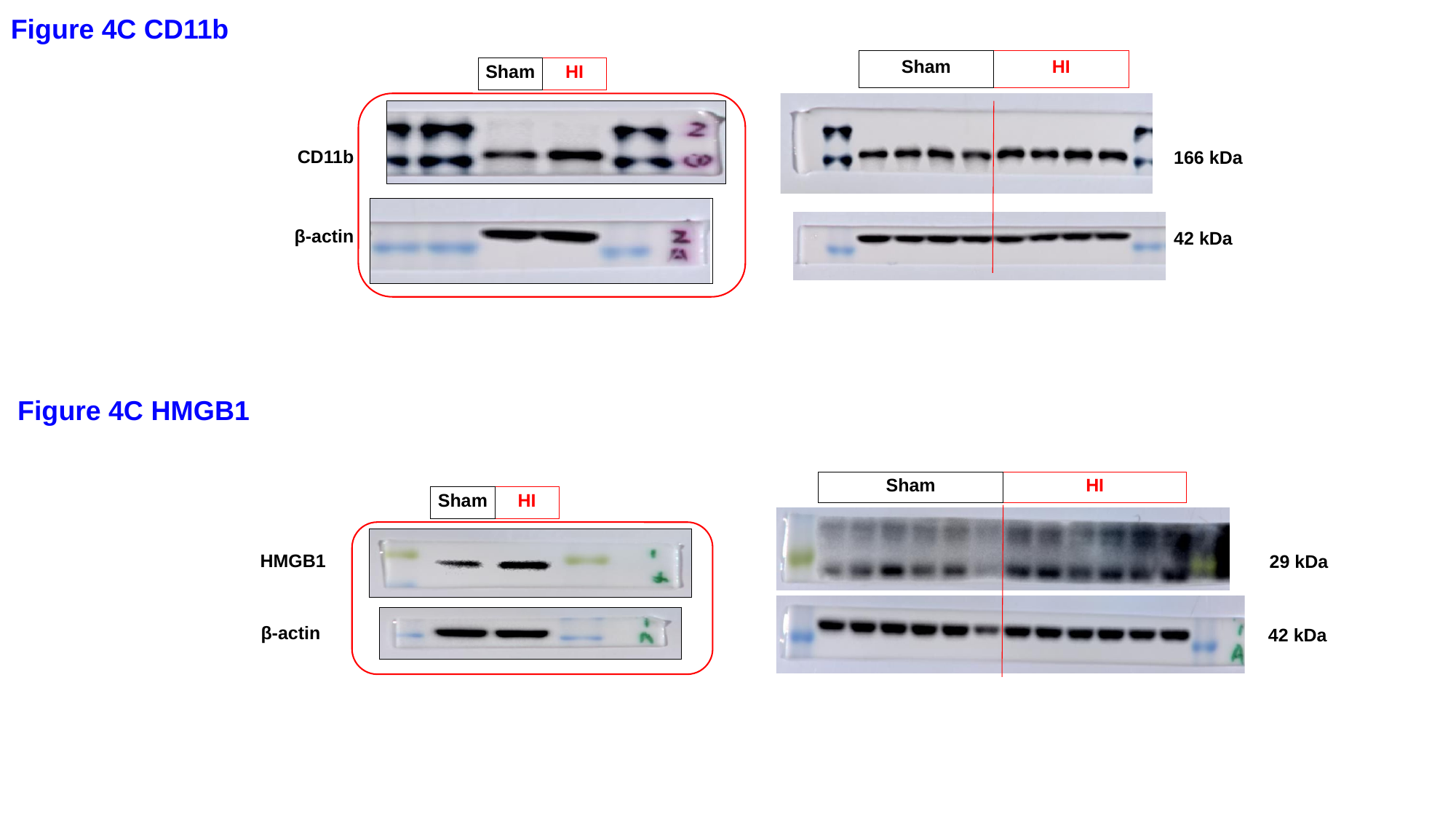

Figure 4C CD11b
| Sham | HI |
| --- | --- |
| Sham | HI |
| --- | --- |
CD11b
166 kDa
β-actin
42 kDa
Figure 4C HMGB1
| Sham | HI |
| --- | --- |
| Sham | HI |
| --- | --- |
HMGB1
29 kDa
β-actin
42 kDa

## Slide 10
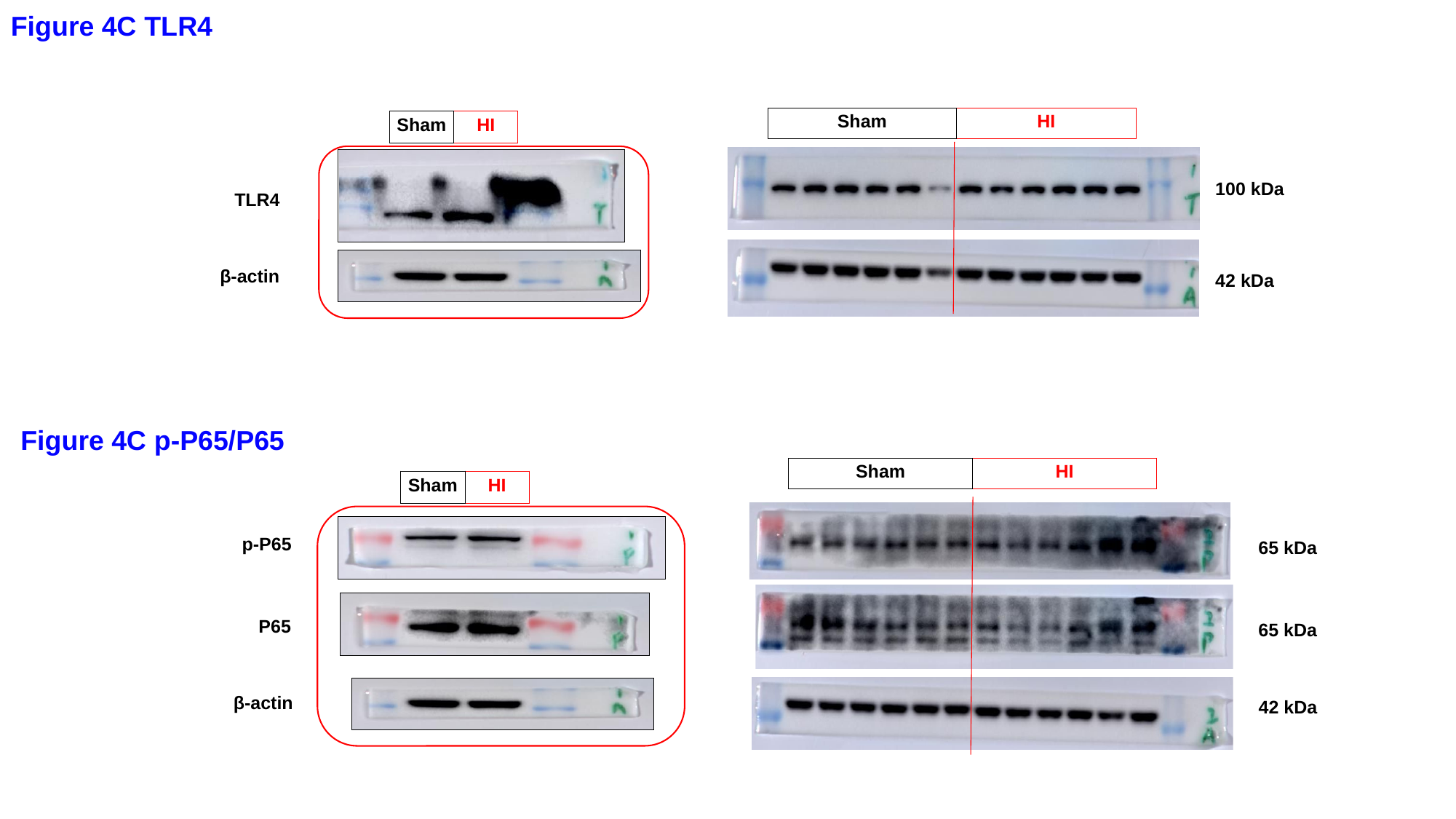

Figure 4C TLR4
| Sham | HI |
| --- | --- |
| Sham | HI |
| --- | --- |
100 kDa
TLR4
β-actin
42 kDa
Figure 4C p-P65/P65
| Sham | HI |
| --- | --- |
| Sham | HI |
| --- | --- |
p-P65
65 kDa
P65
65 kDa
β-actin
42 kDa

## Slide 11
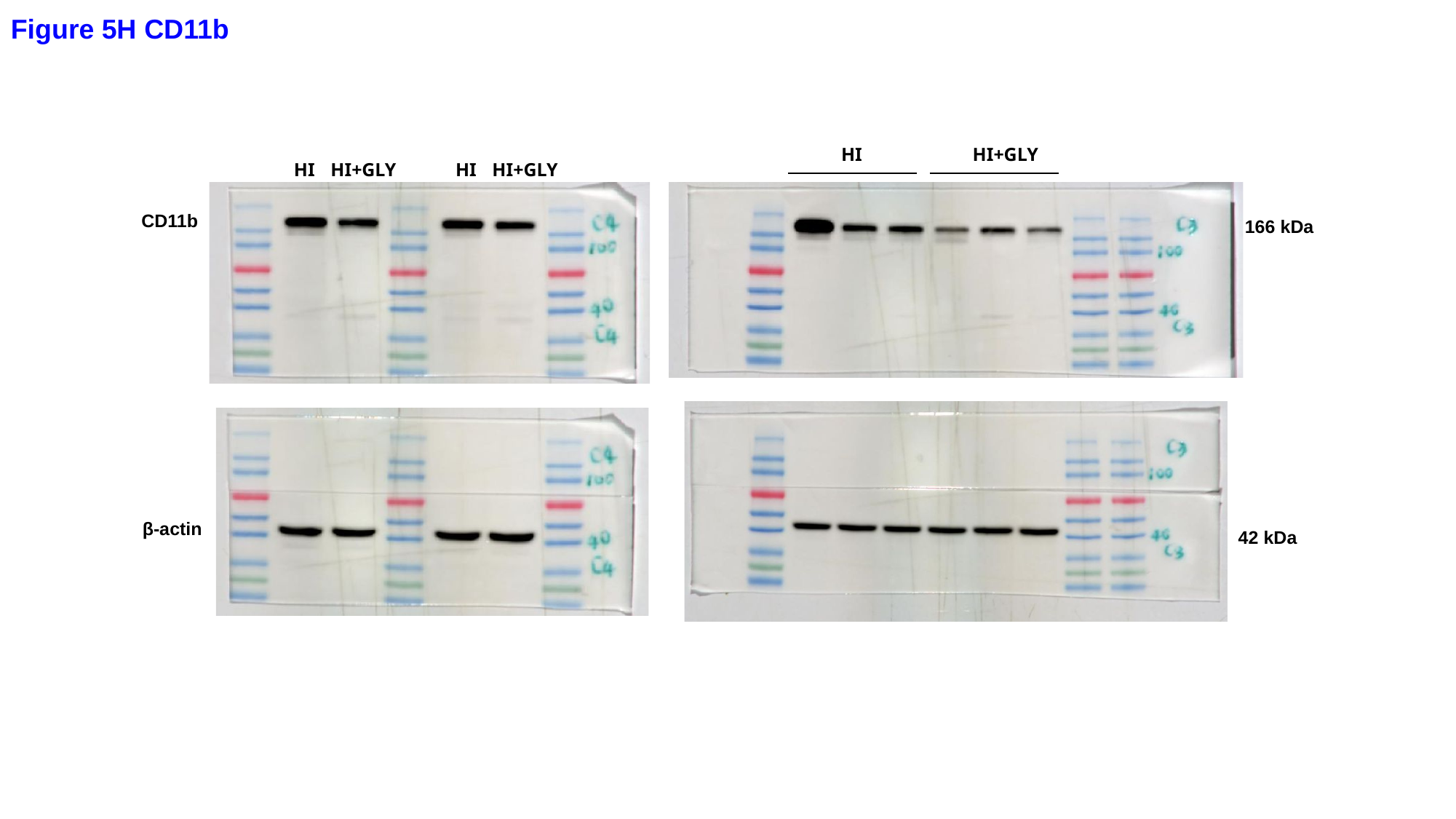

Figure 5H CD11b
HI
HI+GLY
HI
HI+GLY
HI
HI+GLY
CD11b
166 kDa
β-actin
42 kDa

## Slide 12
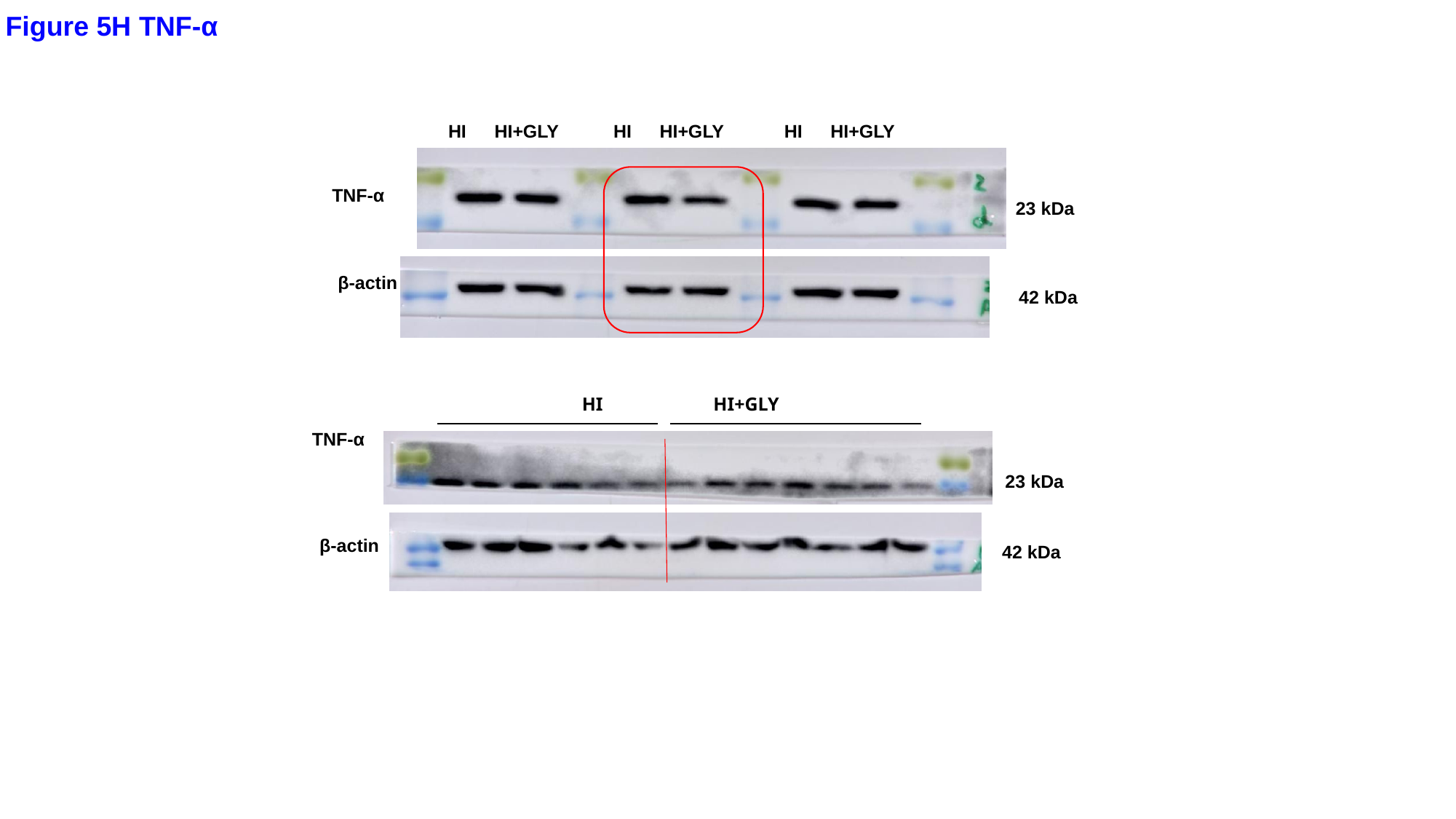

Figure 5H TNF-α
HI
HI+GLY
HI
HI+GLY
HI
HI+GLY
TNF-α
23 kDa
β-actin
42 kDa
HI
HI+GLY
TNF-α
23 kDa
β-actin
42 kDa

## Slide 13
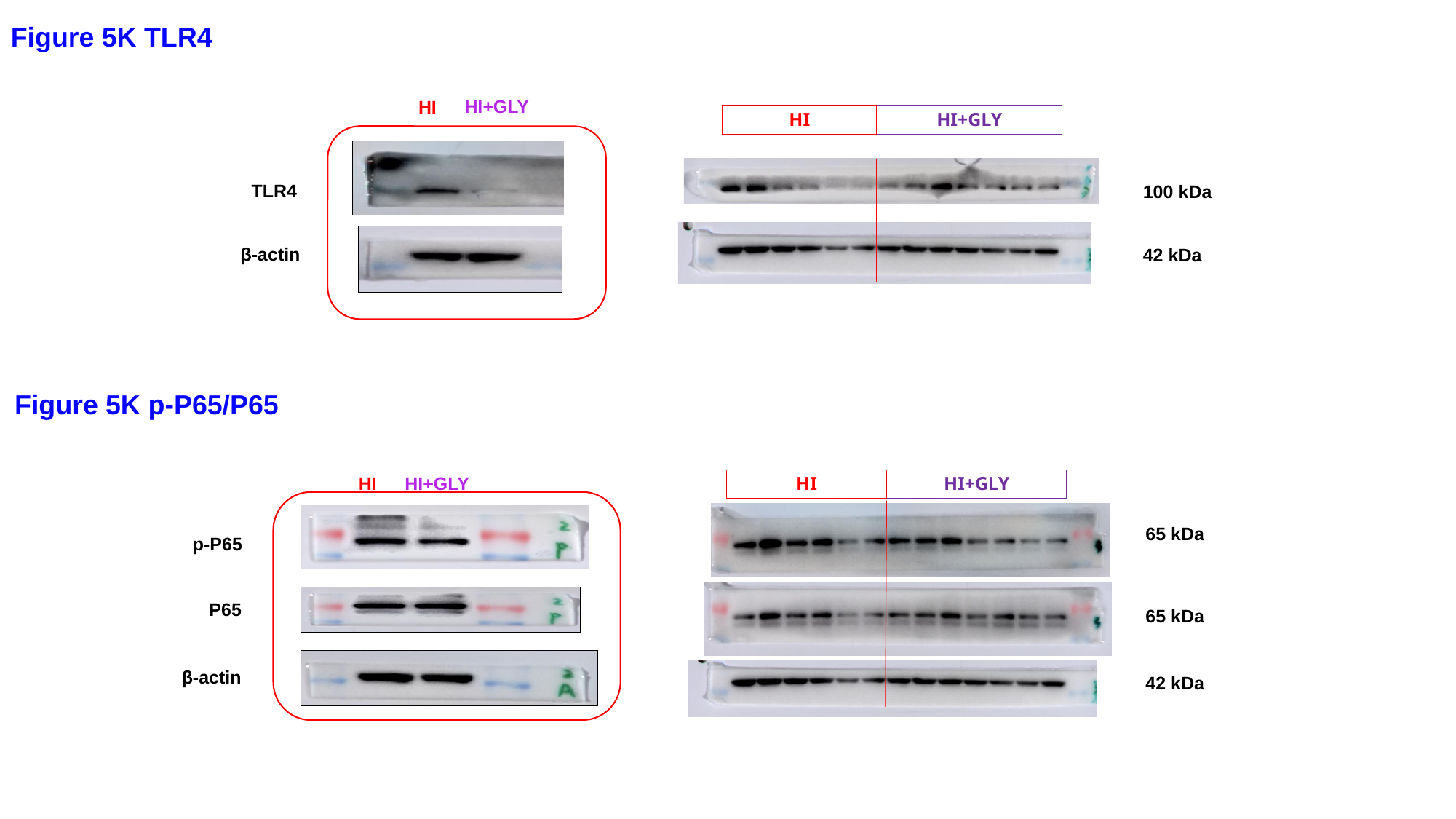

Figure 5K TLR4
HI+GLY
HI
| HI | HI+GLY |
| --- | --- |
TLR4
100 kDa
β-actin
42 kDa
Figure 5K p-P65/P65
HI+GLY
HI
| HI | HI+GLY |
| --- | --- |
65 kDa
p-P65
P65
65 kDa
β-actin
42 kDa

## Slide 14
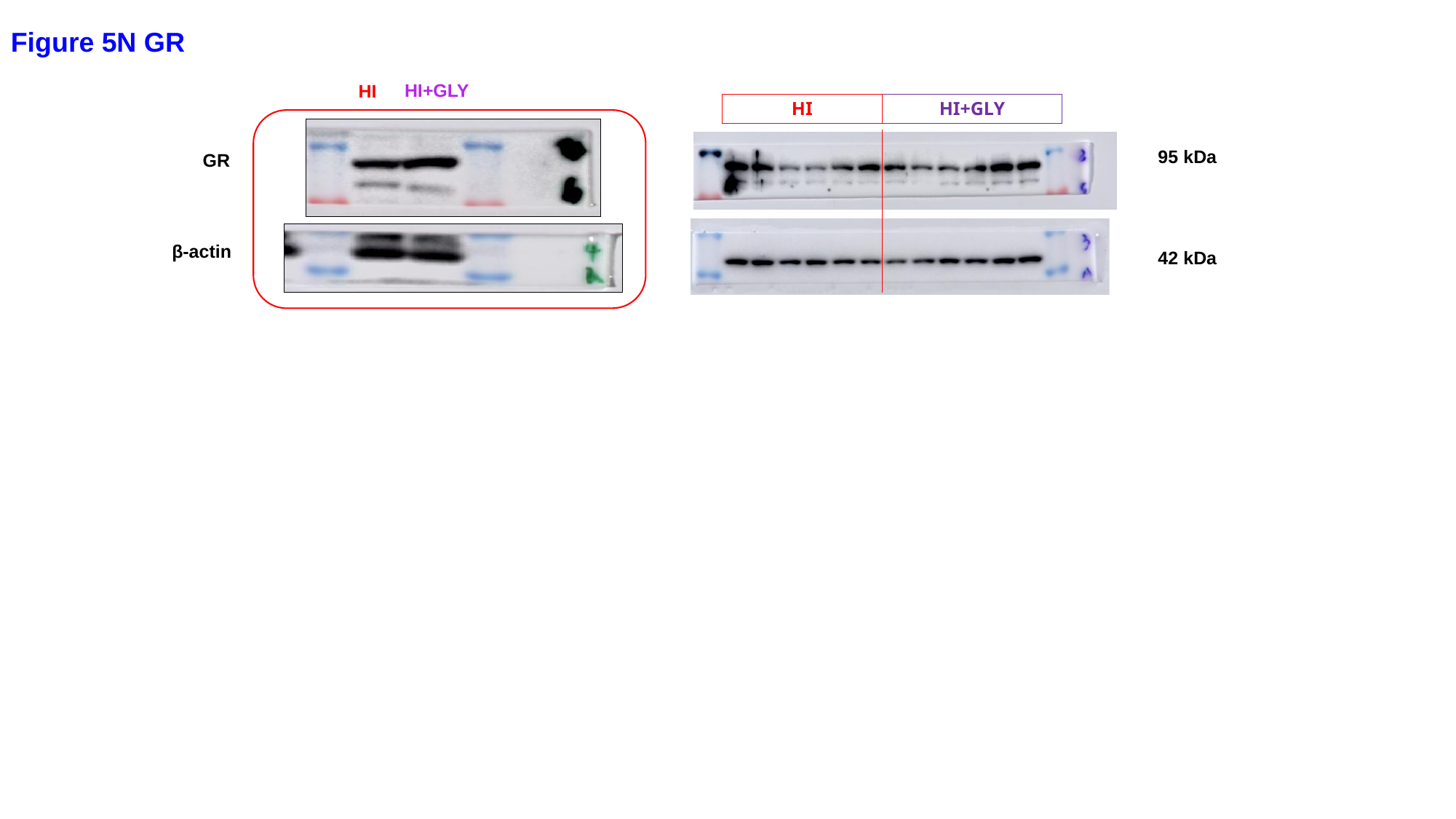

Figure 5N GR
HI+GLY
HI
| HI | HI+GLY |
| --- | --- |
95 kDa
GR
β-actin
42 kDa

## Slide 15
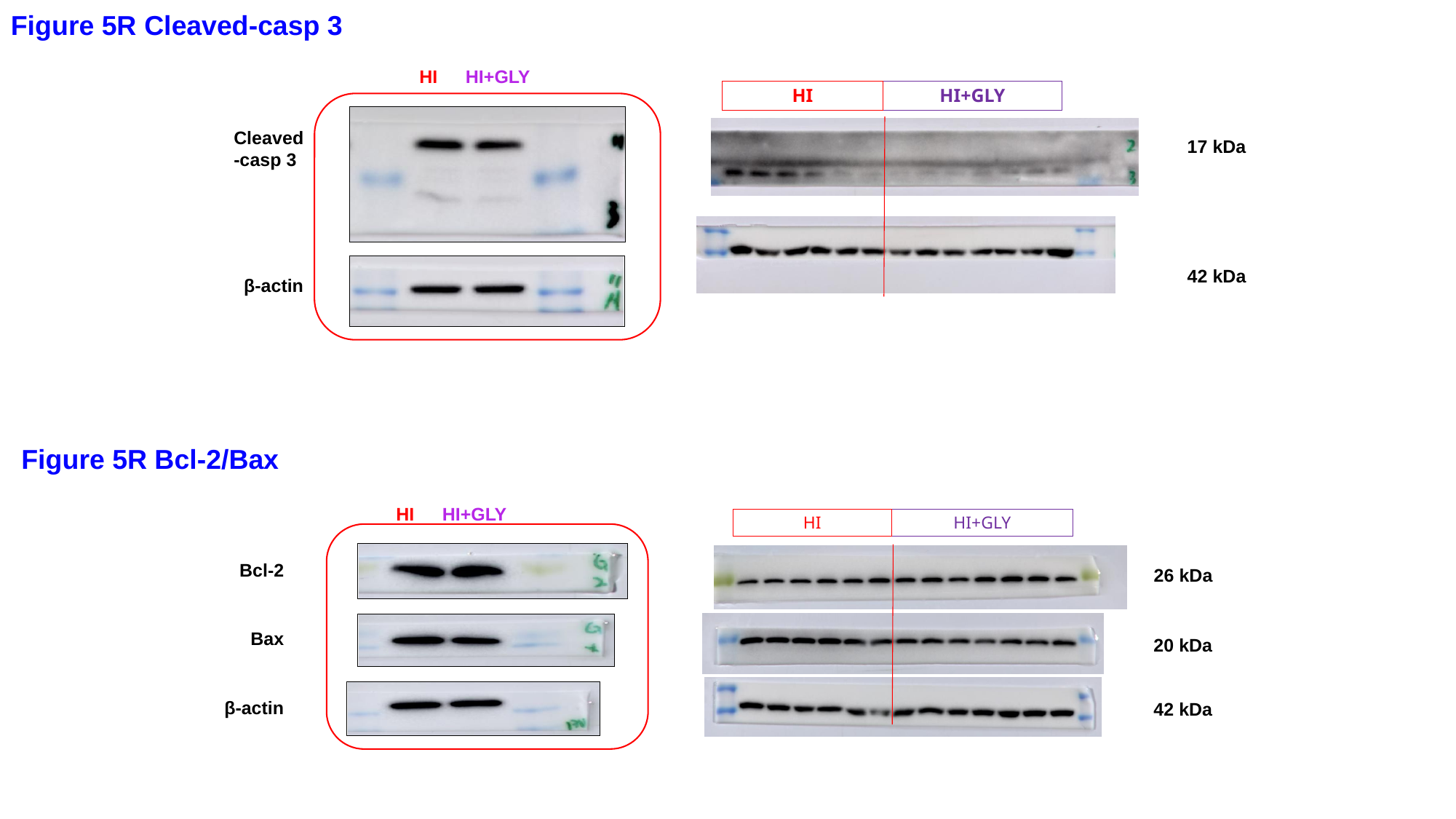

Figure 5R Cleaved-casp 3
HI+GLY
HI
| HI | HI+GLY |
| --- | --- |
Cleaved
-casp 3
17 kDa
42 kDa
β-actin
Figure 5R Bcl-2/Bax
HI+GLY
HI
| HI | HI+GLY |
| --- | --- |
Bcl-2
26 kDa
Bax
20 kDa
β-actin
42 kDa

## Slide 16
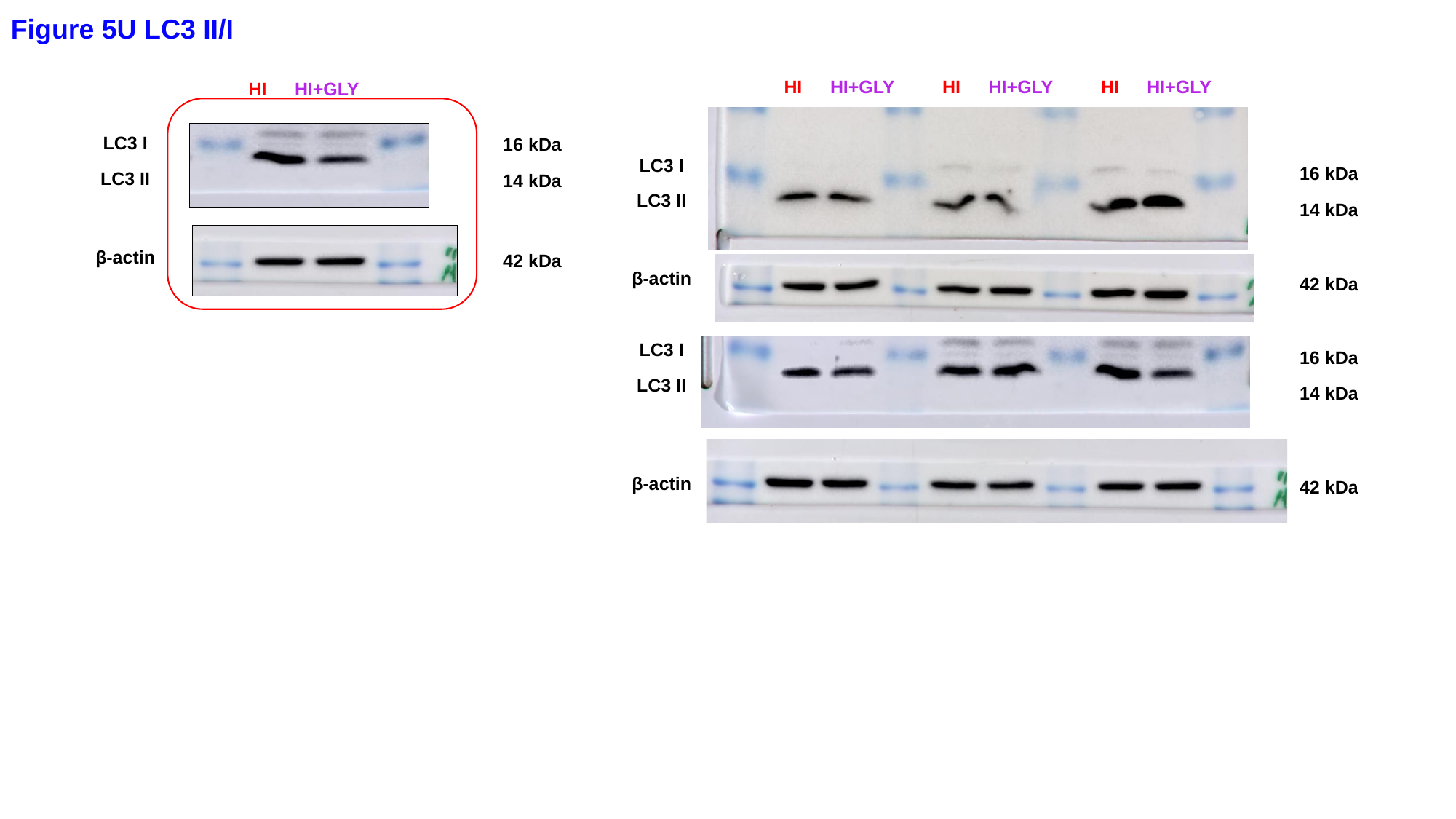

Figure 5U LC3 II/I
HI
HI+GLY
HI
HI+GLY
HI
HI+GLY
HI+GLY
HI
LC3 I
16 kDa
LC3 I
16 kDa
LC3 II
14 kDa
LC3 II
14 kDa
β-actin
42 kDa
β-actin
42 kDa
LC3 I
16 kDa
LC3 II
14 kDa
β-actin
42 kDa

## Slide 17
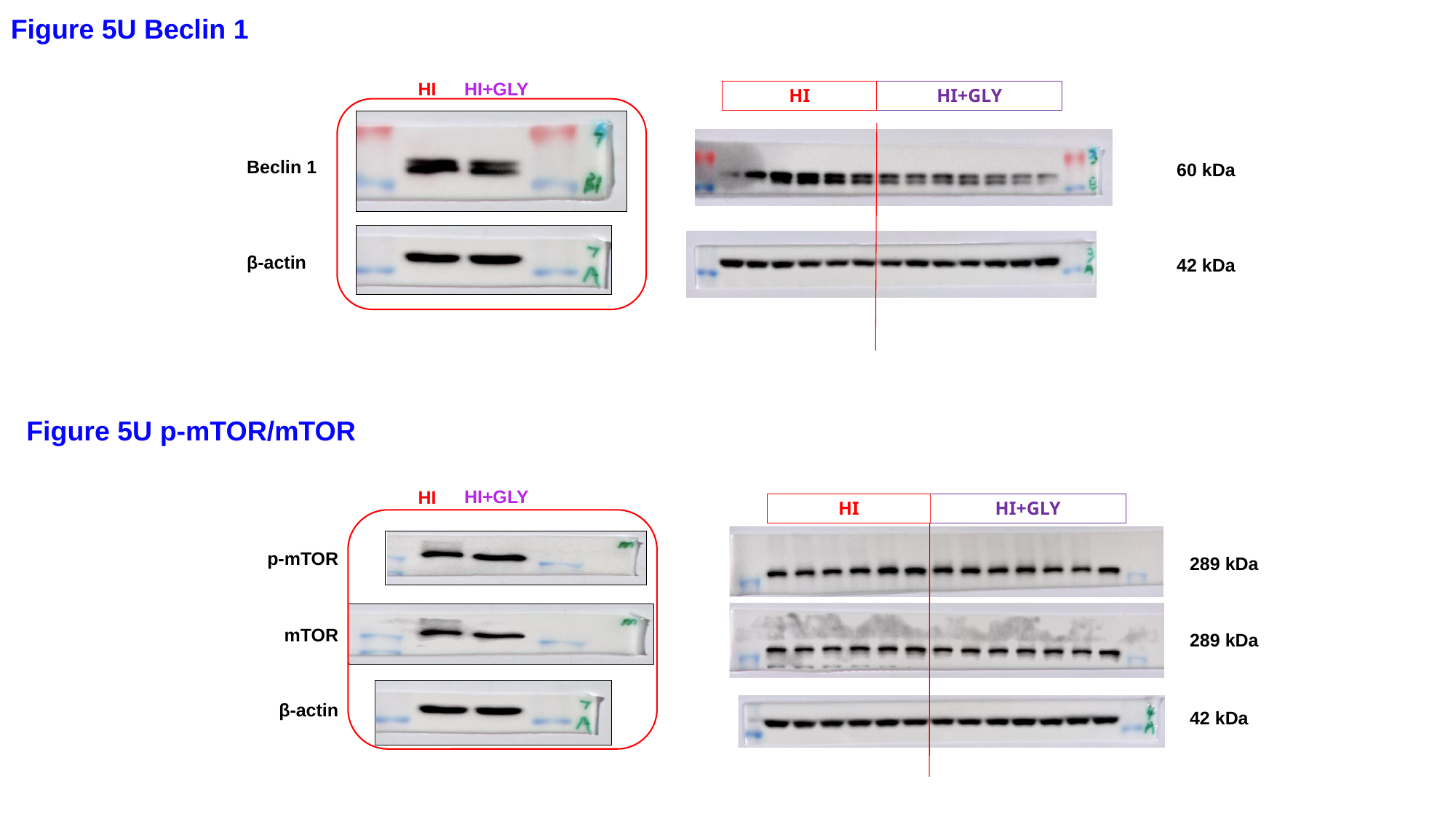

Figure 5U Beclin 1
HI+GLY
HI
| HI | HI+GLY |
| --- | --- |
Beclin 1
60 kDa
β-actin
42 kDa
Figure 5U p-mTOR/mTOR
HI+GLY
HI
| HI | HI+GLY |
| --- | --- |
p-mTOR
289 kDa
mTOR
289 kDa
β-actin
42 kDa

## Slide 18
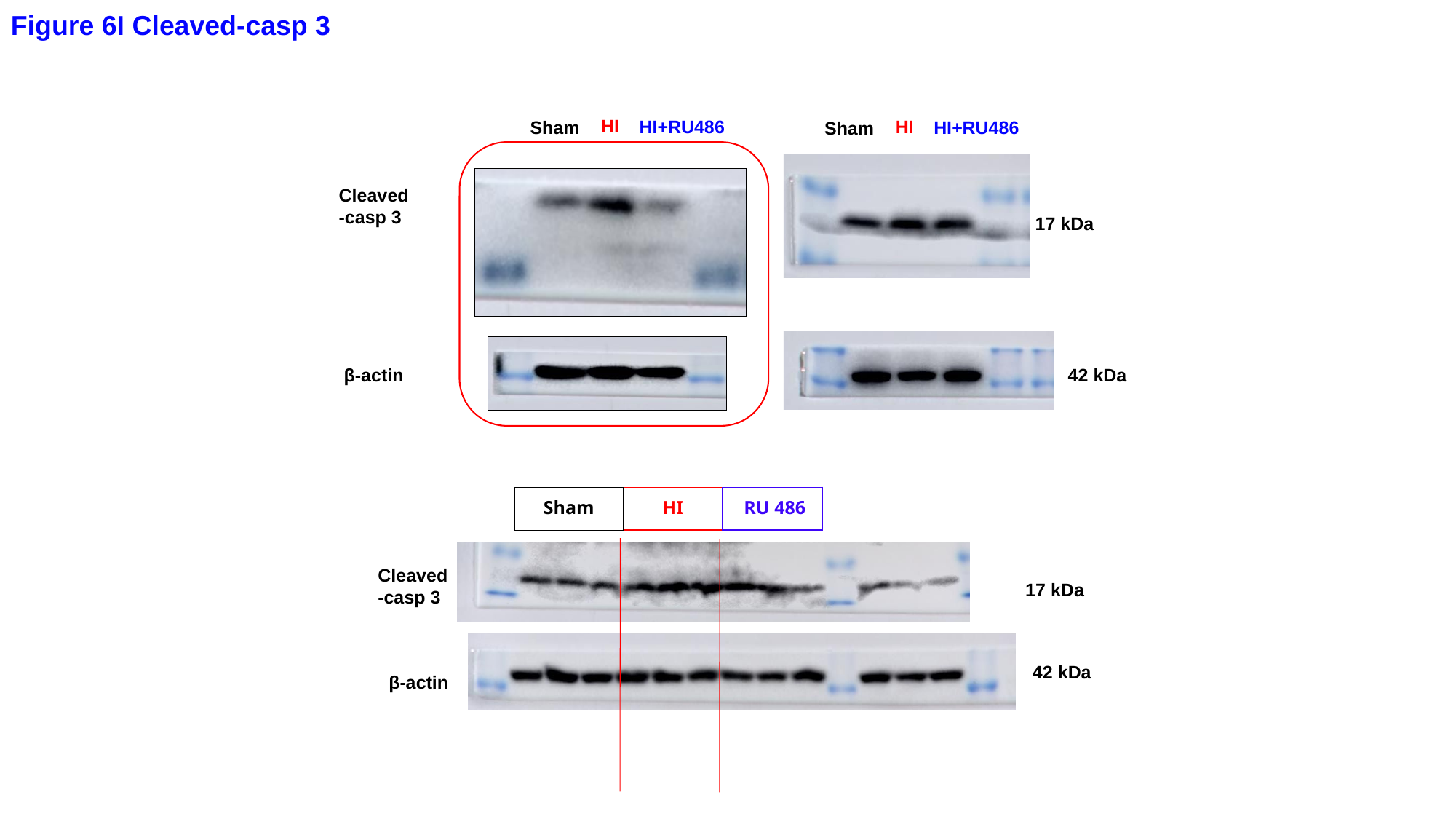

Figure 6I Cleaved-casp 3
HI
HI+RU486
Sham
HI
HI+RU486
Sham
Cleaved
-casp 3
17 kDa
42 kDa
β-actin
| Sham | HI | RU 486 |
| --- | --- | --- |
Cleaved
-casp 3
17 kDa
42 kDa
β-actin

## Slide 19
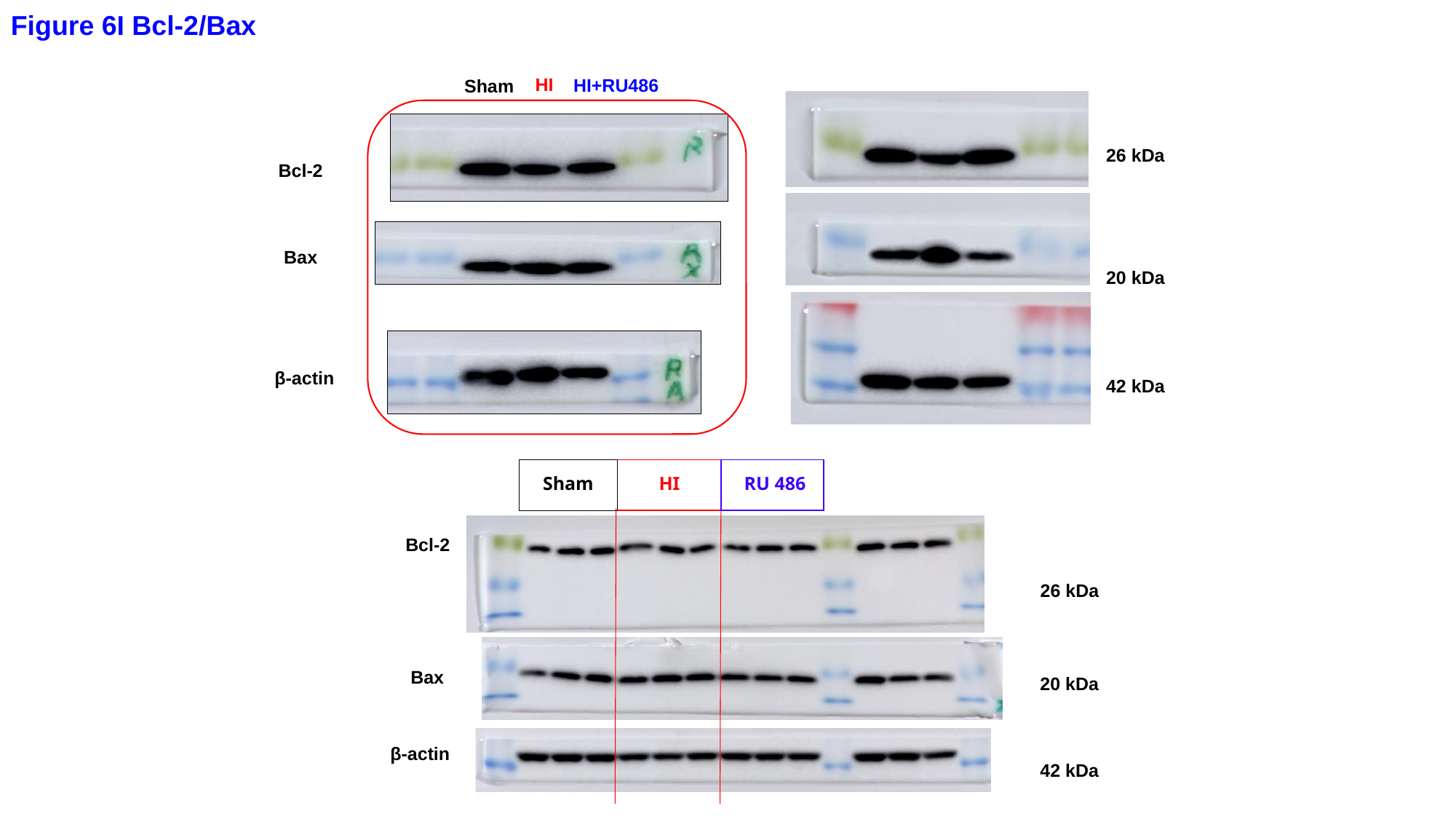

Figure 6I Bcl-2/Bax
HI
HI+RU486
Sham
26 kDa
Bcl-2
Bax
20 kDa
β-actin
42 kDa
| Sham | HI | RU 486 |
| --- | --- | --- |
Bcl-2
26 kDa
Bax
20 kDa
β-actin
42 kDa

## Slide 20
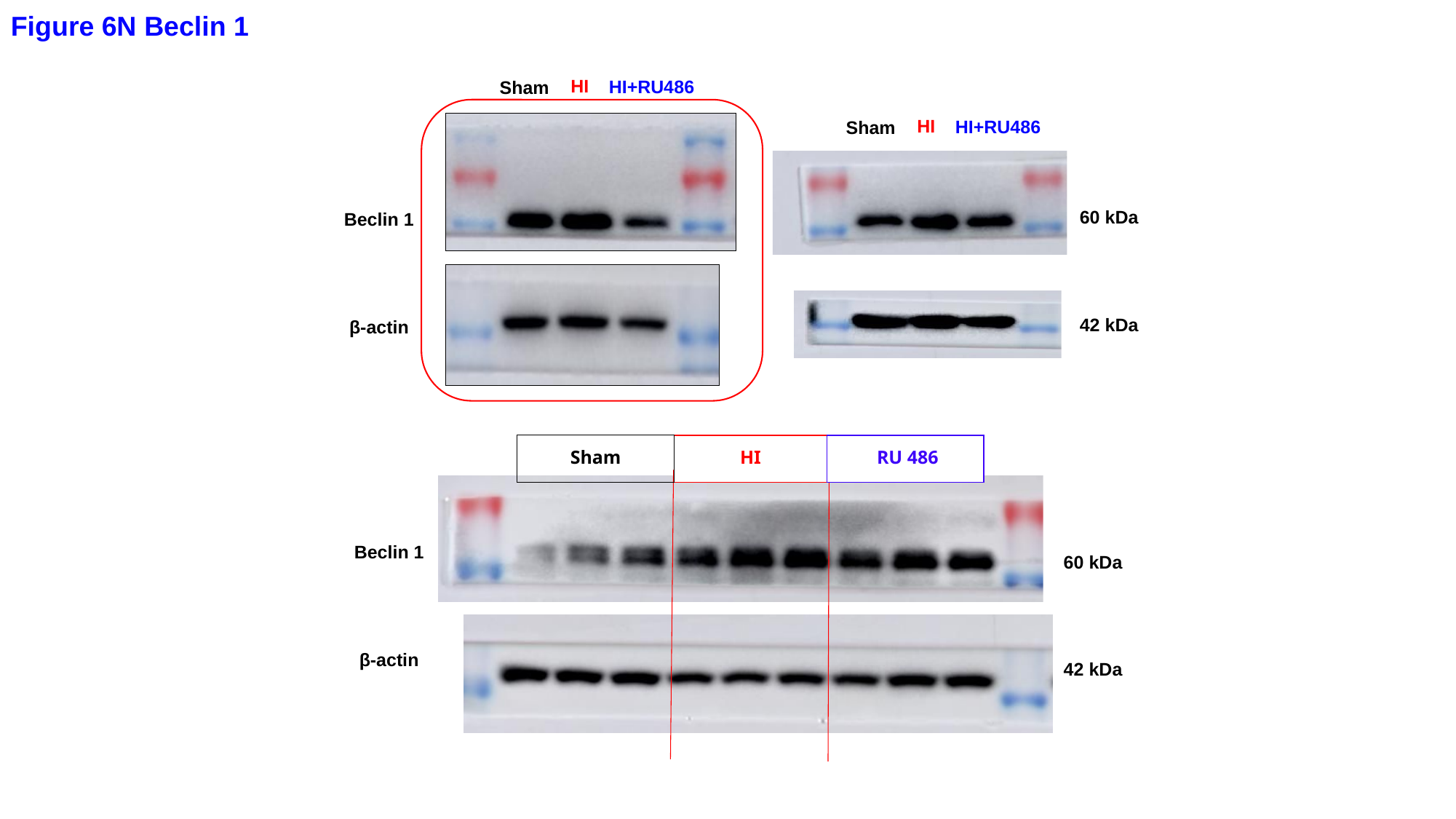

Figure 6N Beclin 1
HI
HI+RU486
Sham
HI
HI+RU486
Sham
60 kDa
Beclin 1
42 kDa
β-actin
| Sham | HI | RU 486 |
| --- | --- | --- |
Beclin 1
60 kDa
β-actin
42 kDa

## Slide 21
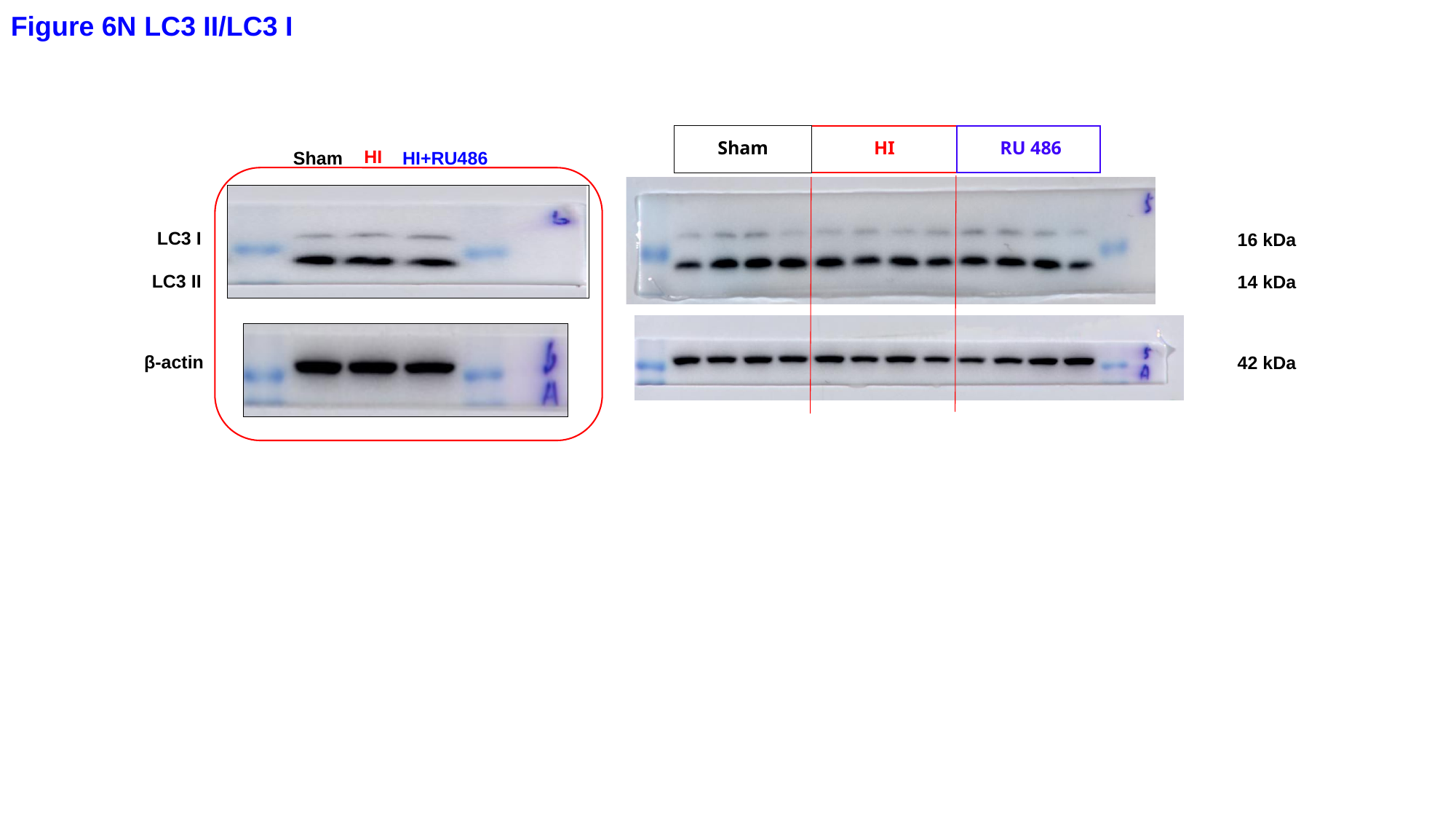

Figure 6N LC3 II/LC3 I
| Sham | HI | RU 486 |
| --- | --- | --- |
HI
HI+RU486
Sham
LC3 I
16 kDa
LC3 II
14 kDa
β-actin
42 kDa

## Slide 22
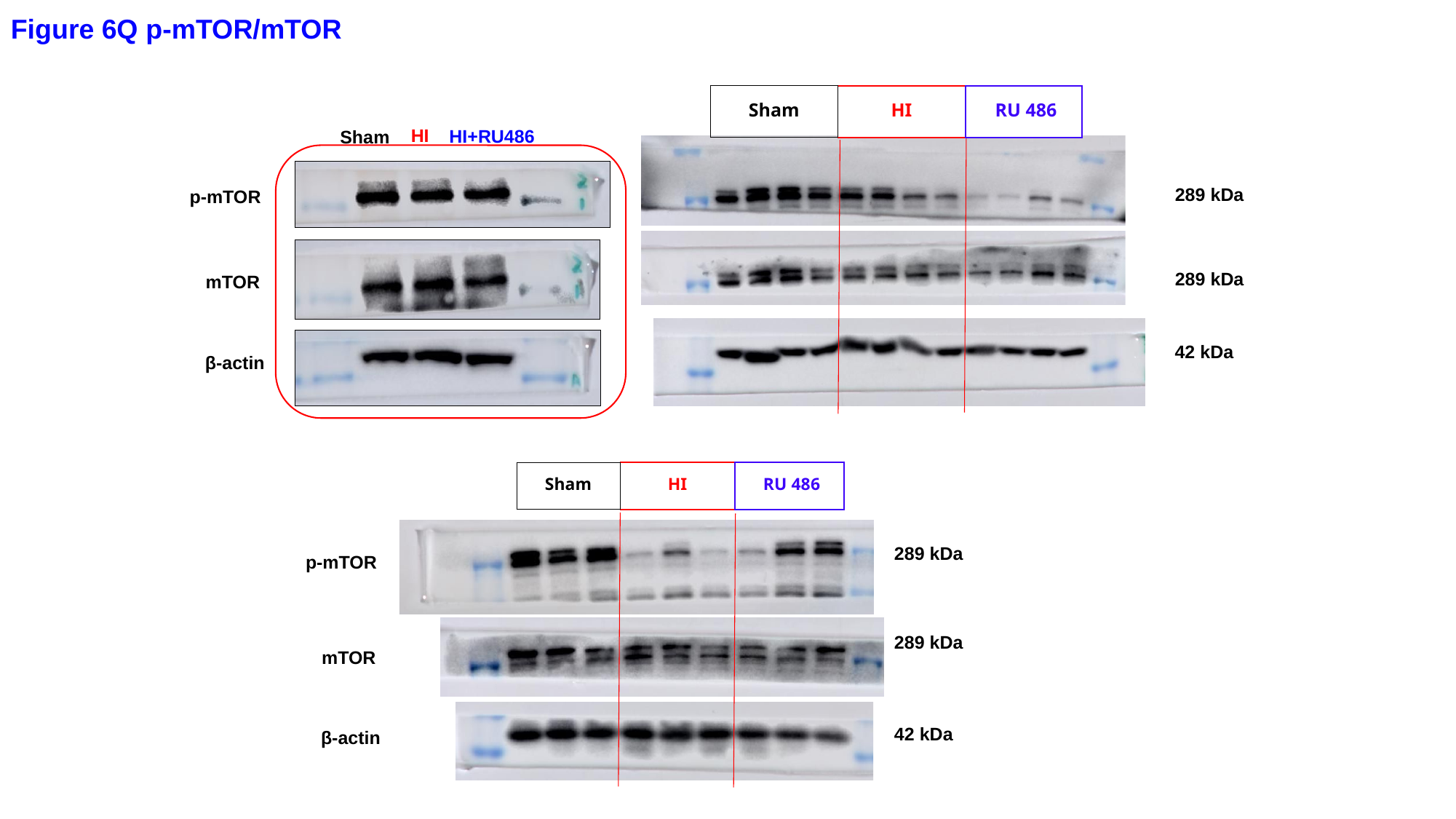

Figure 6Q p-mTOR/mTOR
| Sham | HI | RU 486 |
| --- | --- | --- |
HI
HI+RU486
Sham
289 kDa
p-mTOR
289 kDa
mTOR
42 kDa
β-actin
| Sham | HI | RU 486 |
| --- | --- | --- |
289 kDa
p-mTOR
289 kDa
mTOR
42 kDa
β-actin
